# Supplementary material for: The effect of methylphenidate on anaesthesia recovery: An experimental study in pigs
Source: PLoS One. 2024 Apr 16;19(4):e0302166. doi: 10.1371/journal.pone.0302166 (PMC11020859; doi:10.1371/journal.pone.0302166)

| | BSL | TP5 | TP10 | TP15 |
|---|---|---|---|---|
| **Control** | 110 | 111 | 113 | |
| | 99 | 114 | 116 | 116 |
| | 162 | 160 | 136 | 148 |
| | 193 | 192 | 192 | 175 |
| | 170 | 158 | 134 | 135 |

| | BSL | TP5 | TP10 | TP15 |
|---|---|---|---|---|
| **MP 10 mg/kg** | 123 | 137 | 147 | |
| | 116 | 120 | 116 | 110 |
| | 174 | 184 | 167 | 160 |
| | 157 | 150 | 137 | 134 |
| | 177 | 172 | | |

| | BSL | TP5 | TP10 | TP15 |
|---|---|---|---|---|
| **MP 20 mg/kg** | 135 | 140 | 137 | 148 |
| | 137 | 132 | | |
| | 144 | 159 | 160 | 160 |
| | 200 | 245 | 235 | |
| | 204 | 177 | 166 | 145 |

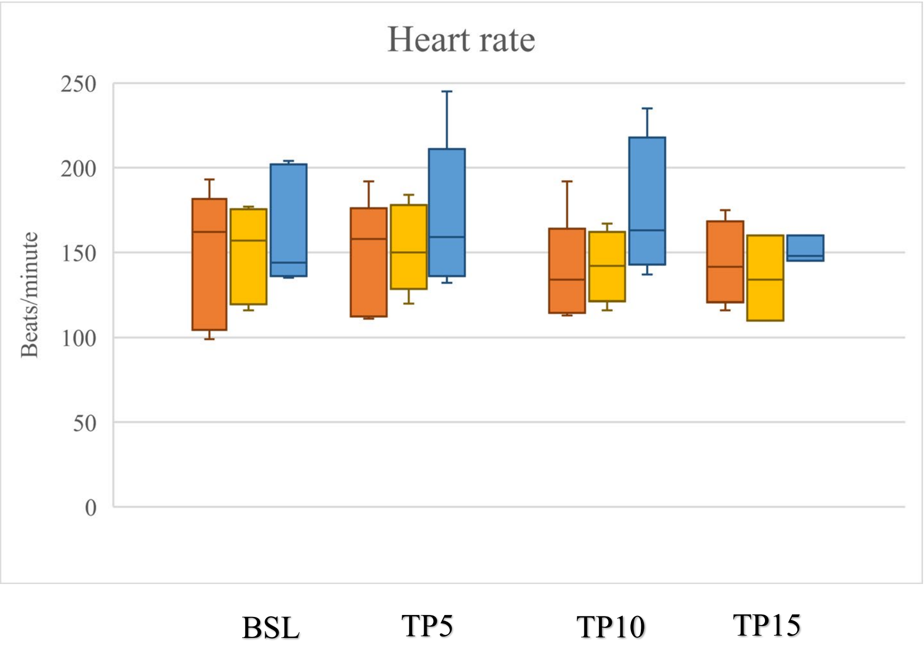

| | BSL | TP5 | TP10 | TP15 |
|---|---|---|---|---|
| **Control** | 16 | 22 | 24 | |
| | 14 | 18 | 20 | 22 |
| | 25 | 29 | 32 | 27 |
| | 19 | 31 | 37 | 32 |
| | 29 | 37 | 37 | 34 |

| | BSL | TP5 | TP10 | TP15 |
|---|---|---|---|---|
| **MP 10 mg/kg** | 21 | 26 | 26 | 27 |
| | 24 | 30 | 36 | 25 |
| | 37 | 37 | 38 | 34 |
| | 16 | 30 | 33 | 31 |
| | 42 | 59 | | |

| | BSL | TP5 | TP10 | TP15 |
|---|---|---|---|---|
| **MP 20 mg/kg** | 17 | 28 | 24 | 33 |
| | 18 | 25 | | |
| | 20 | 27 | 28 | 32 |
| | 17 | 32 | 37 | |
| | 31 | 39 | 40 | 30 |

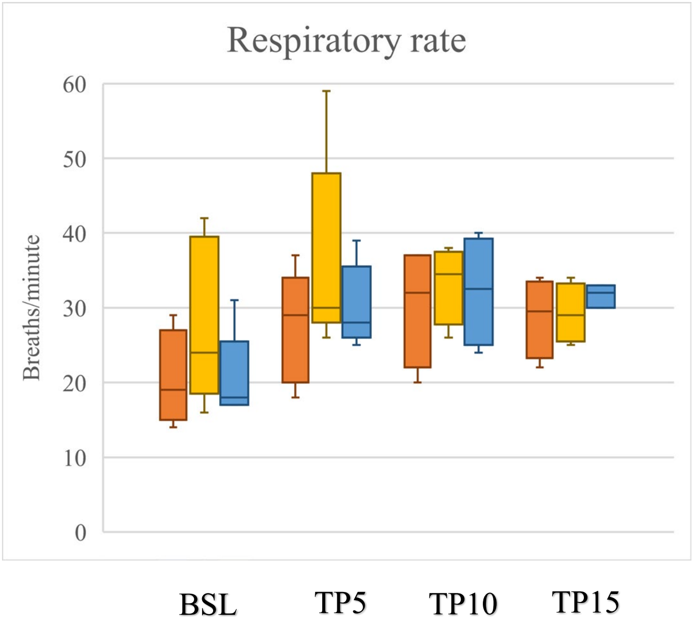

| | BSL | TP5 | TP10 | TP15 |
|---|---|---|---|---|
| **Control** | 93 | 90 | 80 | |
| | 74 | 89 | 87 | 79 |
| | 65 | 73 | 68 | 64 |
| | 69 | 67 | 58 | 59 |
| | 58 | 68 | 64 | 61 |

| | BSL | TP5 | TP10 | TP15 |
|---|---|---|---|---|
| **MP 10 mg/kg** | | 121 | 116 | 107 |
| | 85 | 106 | 100 | 88 |
| | 76 | 84 | 78 | 78 |
| | 61 | 92 | 90 | 90 |
| | 72 | 76 | | |

| | BSL | TP5 | TP10 | TP15 |
|---|---|---|---|---|
| **MP 20 mg/kg** | 101 | 95 | 109 | 105 |
| | 94 | 105 | | |
| | 64 | 87 | 86 | 85 |
| | 137 | 101 | 63 | |
| | 77 | 85 | 81 | 78 |

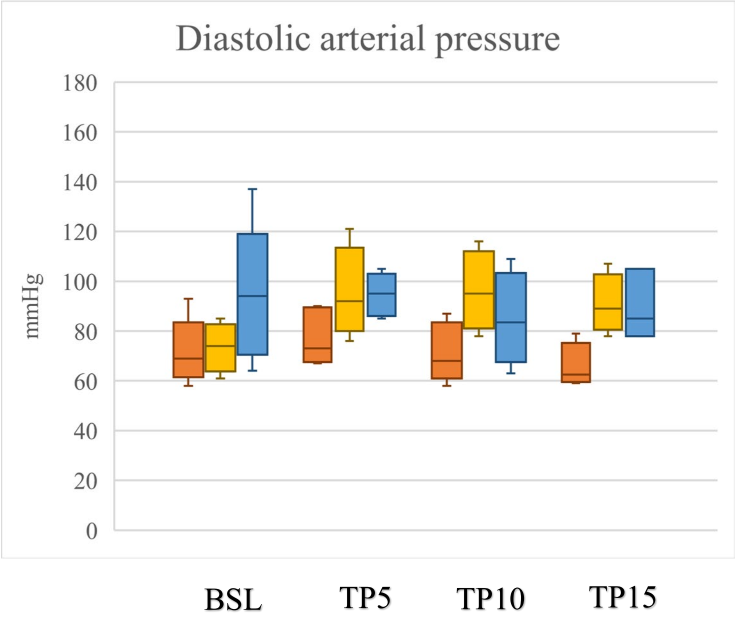

| | BSL | TP5 | TP10 | TP15 |
|---|---|---|---|---|
| **Control** | 146 | 148 | 146 | |
| | 122 | 131 | 124 | 120 |
| | 129 | 135 | 117 | 112 |
| | 126 | 126 | 110 | 105 |
| | 104 | 106 | 104 | 98 |

| | BSL | TP5 | TP10 | TP15 |
|---|---|---|---|---|
| **MP 10 mg/kg** | | 158 | 143 | 130 |
| | 135 | 140 | 134 | 128 |
| | 130 | 131 | 128 | 123 |
| | 110 | 139 | 140 | 139 |
| | 112 | 120 | | |

| | BSL | TP5 | TP10 | TP15 |
|---|---|---|---|---|
| **MP 20 mg/kg** | 140 | 102 | 120 | 144 |
| | 143 | 145 | | |
| | 121 | 134 | 126 | 120 |
| | 141 | 171 | 131 | |
| | 125 | 118 | 118 | 119 |

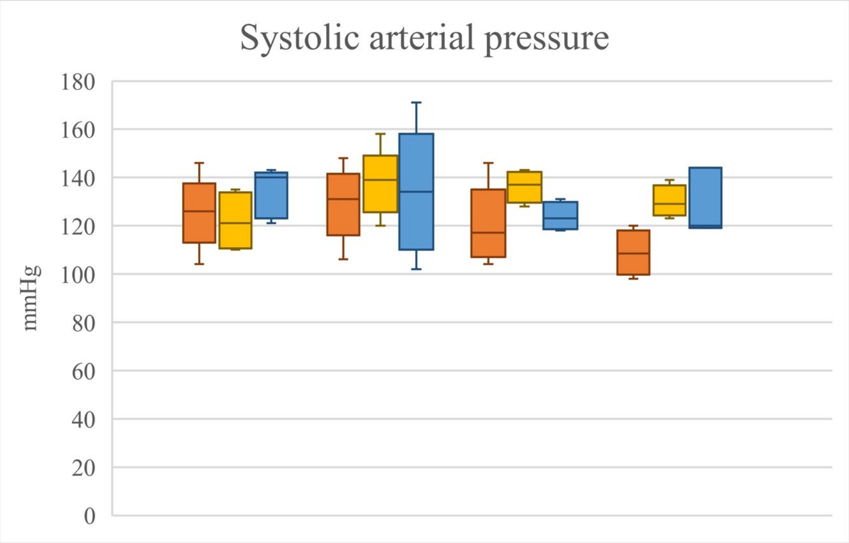


BSL TP5 TP10 TP15

| | BSL | TP5 | TP10 | TP15 |
| --- | --- | --- | --- | --- |
| **Control** | 112 | 111 | 105 | |
| | 98 | 106 | 102 | 95 |
| | 90 | 95 | 88 | 84 |
| | 87 | 87 | 75 | 72 |
| | 76 | 84 | 83 | 78 |

| | BSL | TP5 | TP10 | TP15 |
| --- | --- | --- | --- | --- |
| **MP 10 mg/kg** | | 135 | 128 | 118 |
| | 106 | 120 | 114 | 104 |
| | 93 | 99 | 97 | 91 |
| | 83 | 110 | 110 | 109 |
| | 87 | 92 | | |

| | BSL | TP5 | TP10 | TP15 |
| --- | --- | --- | --- | --- |
| **MP 20 mg/kg** | 117 | 105 | 123 | 120 |
| | 111 | 120 | | |
| | 89 | 105 | 101 | 95 |
| | 139 | 120 | 89 | |
| | 92 | 98 | 95 | 92 |

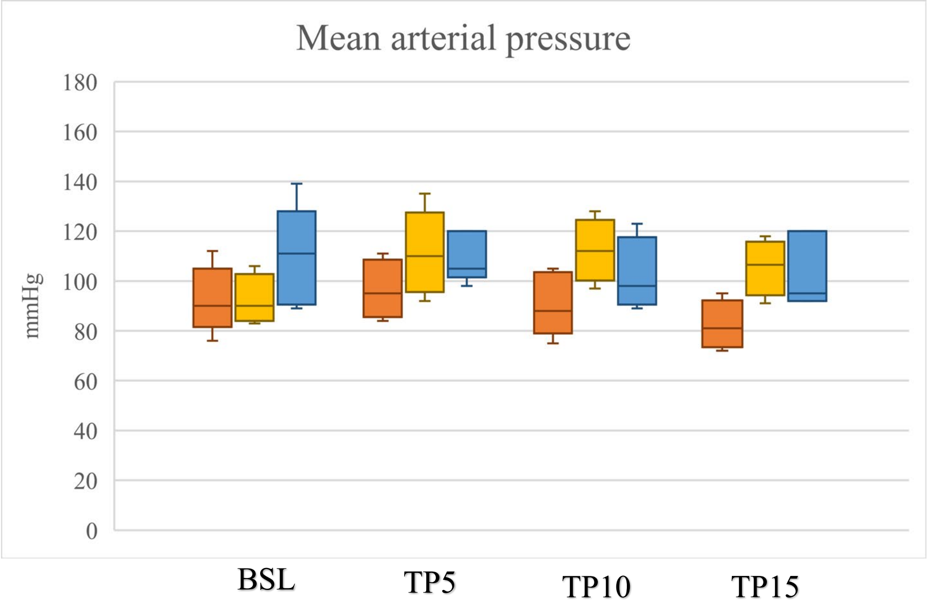

| | Palpebral | Jaw Tone | NWR baseline | Extubation |
|---|---|---|---|---|
| **Control** | 8 | 13 | 18 | 12 |
| | 22 | 14 | 8 | 22 |
| | 7 | 7 | 40 | 19 |
| | 15 | 23 | | 24 |
| | 11 | 18 | 21 | 18 |

| | Palpebral | Jaw Tone | NWR baseline | Extubation |
|---|---|---|---|---|
| **MP 10 mg/kg** | 5 | 20 | 14 | 15 |
| | | | 3 | 20 |
| | 8 | 20 | 28 | 23 |
| | 15 | 15 | | 22 |
| | 1 | 8 | 30 | 10 |

| | Palpebral | Jaw Tone | NWR baseline | Extubation |
|---|---|---|---|---|
| **MP 20 mg/kg** | 10 | 30 | 9 | 37 |
| | 26 | 26 | 28 | 33 |
| | 15 | 25 | | 20 |
| | 3 | 30 | 15 | 15 |
| | 6 | 30 | 57 | 22 |

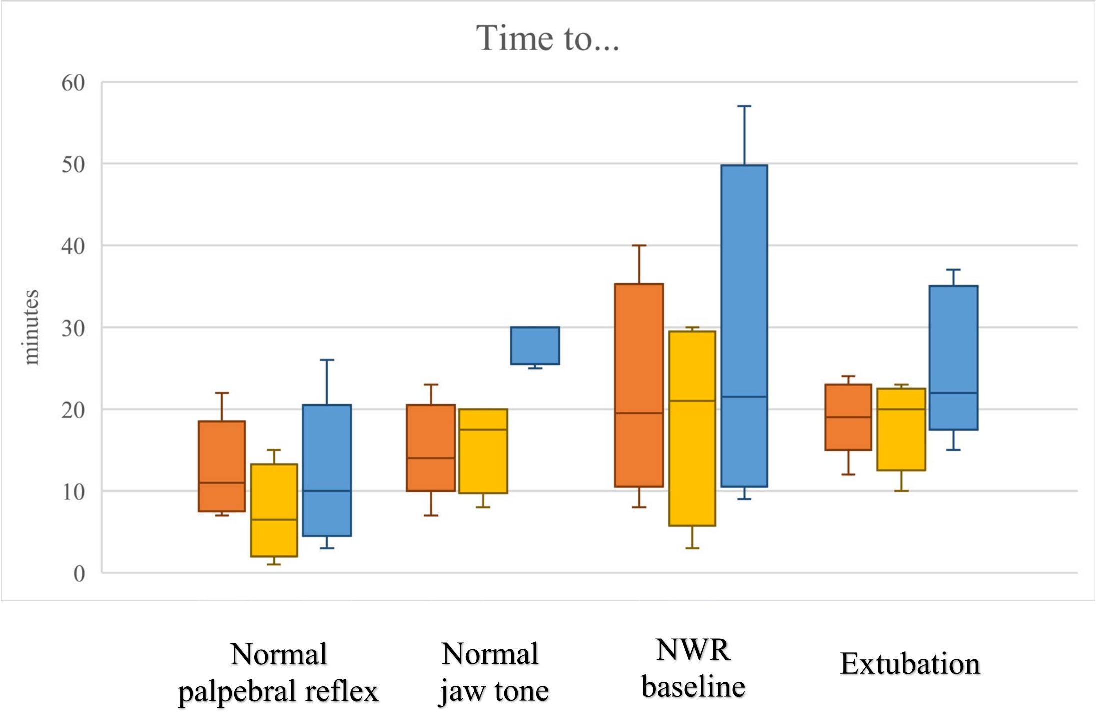

| | BSL | TP5 | TP10 | TP15 |
|---|---|---|---|---|
| **Control** | 28.7 | 20.6 | 17.9 | |
| | 30.6 | 29.8 | 12.8 | 3.9 |
| | 26.5 | 17.5 | 15.4 | 11.8 |
| | 111.8 | 132.3 | 15.0 | |
| | 12.1 | 11.7 | 9.1 | 8.8 |

| | BSL | TP5 | TP10 | TP15 |
|---|---|---|---|---|
| **MP 10 mg/kg** | 37.6 | 37.5 | 23.3 | 16.3 |
| | 22.9 | 18.5 | 8.0 | 2.8 |
| | 24.6 | 28.3 | 17.6 | 12.8 |
| | 33.2 | 32.7 | 29.4 | 19.5 |
| | 37.3 | 38.8 | | |

| | BSL | TP5 | TP10 | TP15 |
|---|---|---|---|---|
| **MP 20 mg/kg** | 22.6 | 15.8 | 17.4 | 5.0 |
| | 26.3 | 19.2 | 16.5 | 16.1 |
| | 35.9 | 42.5 | 39.6 | 35.6 |
| | 70.0 | 111.1 | | 13.6 |
| | 19.2 | 16.9 | 17.8 | 19.3 |

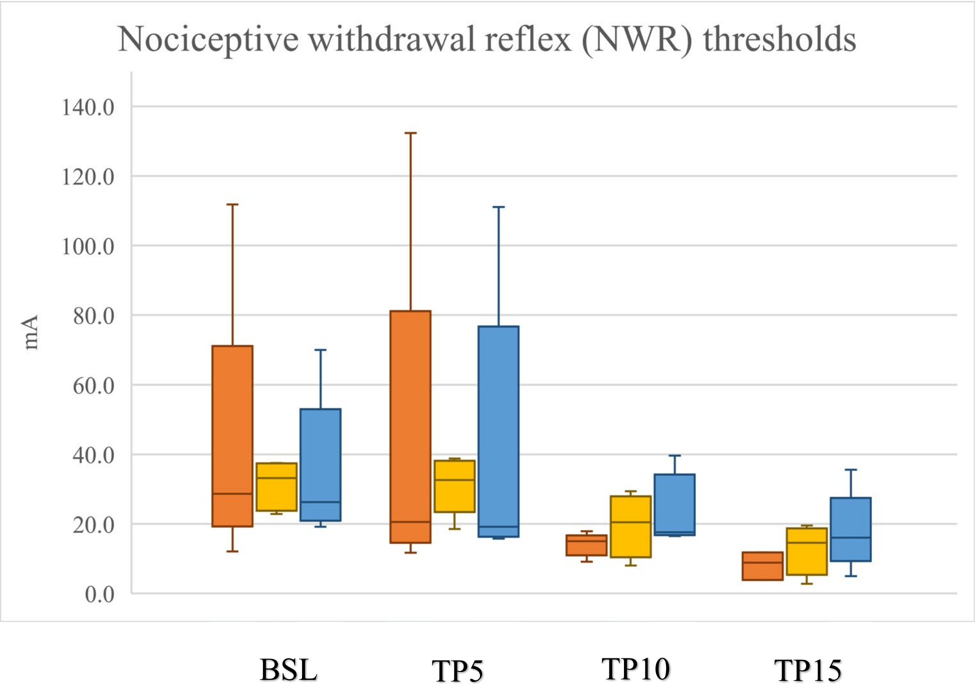

| | PSI back to… | | | |
|---|---|---|---|---|
| | **50** | **60** | **70** | **80** |
| **Control** | 13 | 15 | 27 | 62 |
| | | 21 | 56 | 98 |
| | 17 | 26 | 35 | 81 |
| | 12 | 15 | 24 | 64 |
| | 17 | 19 | 29 | 59 |

| | PSI back to… | | | |
|---|---|---|---|---|
| | **50** | **60** | **70** | **80** |
| **MP 10 mg/kg** | 22 | 24 | 37 | 59 |
| | | | | |
| | 18 | 21 | 24 | 56 |
| | 17 | 19 | 21 | 49 |
| | 6 | 7 | 8 | 27 |

| | PSI back to… | | | |
|---|---|---|---|---|
| | **50** | **60** | **70** | **80** |
| **MP 20 mg/kg** | 29 | 33 | 39 | 64 |
| | 18 | 24 | 27 | 46 |
| | 17 | 19 | 24 | 62 |
| | 13 | 15 | 15 | 16 |
| | 19 | 20 | 22 | 30 |

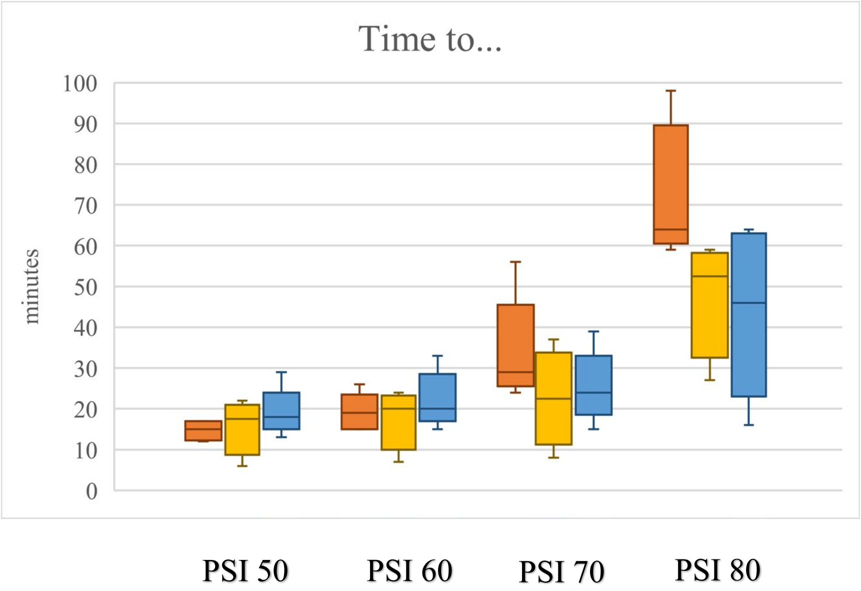

| | BSL | TP5 | TP10 | TP15 |
|---|---|---|---|---|
| Control | 40.0 | 43.3 | 40.3 | |
| | 21.0 | 42.0 | 45.0 | 49.0 |
| | 20.7 | 38.9 | 40.1 | 46.7 |
| | 19.9 | 34.8 | 34.1 | 61.6 |
| | 22.3 | 37.3 | 39.6 | 46.8 |

| | BSL | TP5 | TP10 | TP15 |
|---|---|---|---|---|
| MP 20 mg/kg | 22.4 | 39.1 | 36.6 | 41.7 |
| | 19.7 | 32.0 | 38.8 | 45.0 |
| | 17.3 | 25.7 | 31.4 | 45.0 |
| | 69.2 | 34.5 | 38.5 | 63.6 |
| | 22.1 | 26.6 | 23.2 | 30.0 |

| | BSL | TP5 | TP10 | TP15 |
|---|---|---|---|---|
| MP 10 mg/kg | 22.4 | 42.2 | 44.1 | 47.3 |
| | | | | |
| | 18.4 | 23.5 | 33.0 | 43.1 |
| | 16.0 | 32.5 | 41.3 | 46.8 |
| | 32.9 | 44.4 | | |

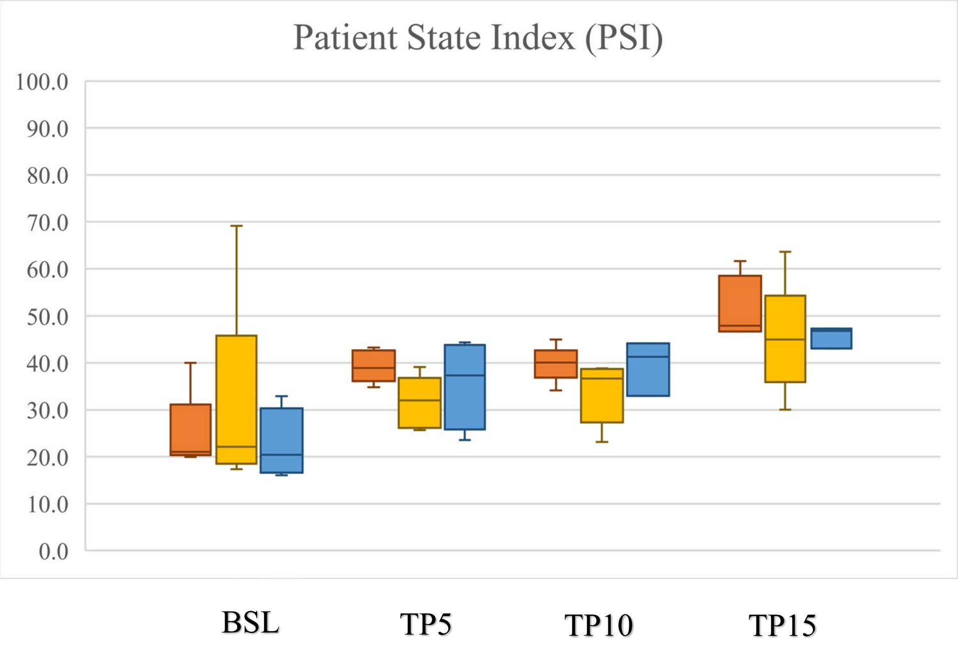

| | BSL | TP5 | TP10 | TP15 |
|---|---|---|---|---|
| **Control** | 0.0 | 0.0 | 0.1 | |
| | 20.0 | 0.0 | 0.0 | 0.0 |
| | 19.6 | 0.0 | 0.0 | 0.0 |
| | 19.6 | 0.0 | 0.0 | 0.0 |
| | 11.8 | 0.0 | 0.0 | 0.0 |

| | BSL | TP5 | TP10 | TP15 |
|---|---|---|---|---|
| **MP 10 mg/kg** | 9.5 | 0.0 | 0.0 | 0.0 |
| | | | | |
| | 28.9 | 0.0 | 0.0 | 0.0 |
| | 33.3 | 0.0 | 0.0 | 0.0 |
| | 0.0 | 0.0 | | |

| | BSL | TP5 | TP10 | TP15 |
|---|---|---|---|---|
| **MP 20 mg/kg** | 11.8 | 0.0 | 0.0 | 0.0 |
| | 23.5 | 0.0 | 0.0 | 0.0 |
| | 31.0 | 0.0 | 0.0 | 0.0 |
| | 0.0 | 0.0 | 0.0 | 0.0 |
| | 10.5 | 13.2 | 0.0 | 0.0 |

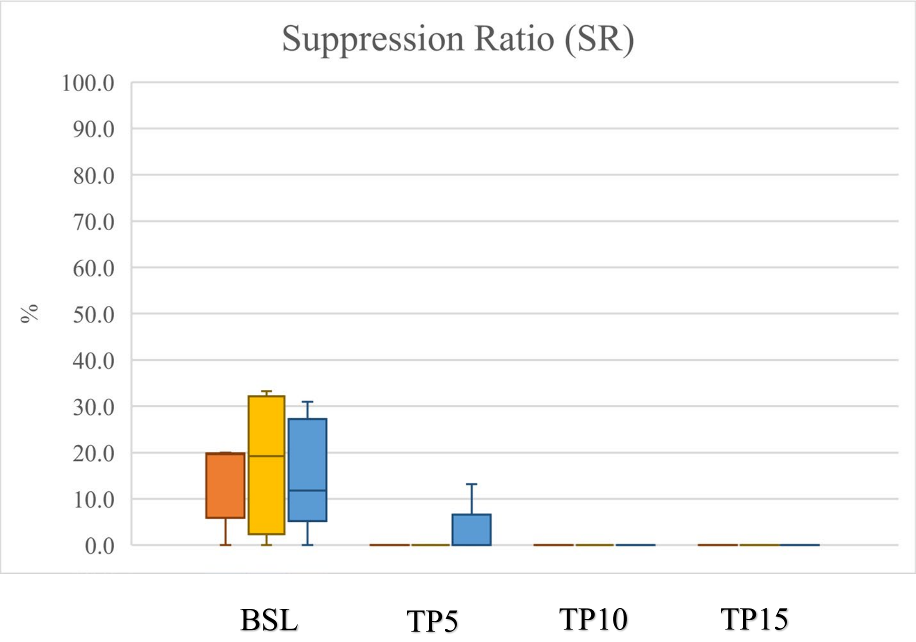

| | BSL | TP5 | TP10 | TP15 |
|---|---|---|---|---|
| **Control** | 15.7 | 18.6 | 16.3 | |
| | 15.4 | 16.6 | 19.9 | 14.5 |
| | 17.2 | 15.6 | 13.6 | 14.4 |
| | 19.7 | 14.1 | 13.7 | 21.3 |
| | 15.5 | 13.4 | 13.0 | 14.7 |

| | BSL | TP5 | TP10 | TP15 |
|---|---|---|---|---|
| **MP 10 mg/kg** | 14.7 | 15.6 | 14.6 | 15.6 |
| | | | | |
| | 15.3 | 12.4 | 11.8 | 13.6 |
| | 14.4 | 13.5 | 13.9 | 14.5 |
| | 14.1 | 13.7 | | |

| | BSL | TP5 | TP10 | TP15 |
|---|---|---|---|---|
| **MP 20 mg/kg** | 18.8 | 18.0 | 15.7 | 15.6 |
| | 17.4 | 14.2 | 15.7 | 16.4 |
| | 16.9 | 13.2 | 12.9 | 15.8 |
| | 16.4 | 13.0 | 14.0 | 17.7 |
| | 8.1 | 10.0 | 7.5 | 8.6 |

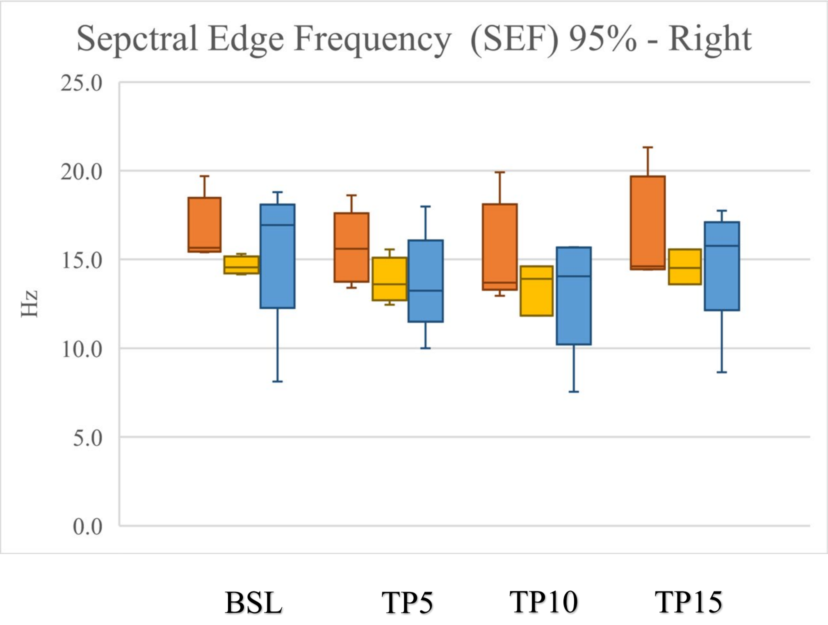

| | BSL | TP5 | TP10 | TP15 |
|---|---|---|---|---|
| **Control** | 17.3 | 19.8 | 17.0 | |
| | 15.6 | 14.9 | 16.7 | 17.3 |
| | 16.6 | 15.2 | 13.4 | 15.3 |
| | 17.6 | 12.2 | 10.7 | 17.3 |
| | 19.3 | 15.0 | 13.8 | 16.6 |

| | BSL | TP5 | TP10 | TP15 |
|---|---|---|---|---|
| **MP 10 mg/kg** | 16.5 | 16.0 | 16.4 | 16.5 |
| | | | | |
| | 16.1 | 11.8 | 11.1 | 13.1 |
| | 17.4 | 15.1 | 14.5 | 15.8 |
| | 14.1 | 13.4 | | |

| | BSL | TP5 | TP10 | TP15 |
|---|---|---|---|---|
| **MP 20 mg/kg** | 16.4 | 16.5 | 14.2 | 13.5 |
| | 16.7 | 12.4 | 15.1 | 16.0 |
| | 14.2 | 13.4 | 13.3 | 15.4 |
| | 23.4 | 11.9 | 12.2 | 14.2 |
| | 13.1 | 16.4 | 12.4 | 12.2 |

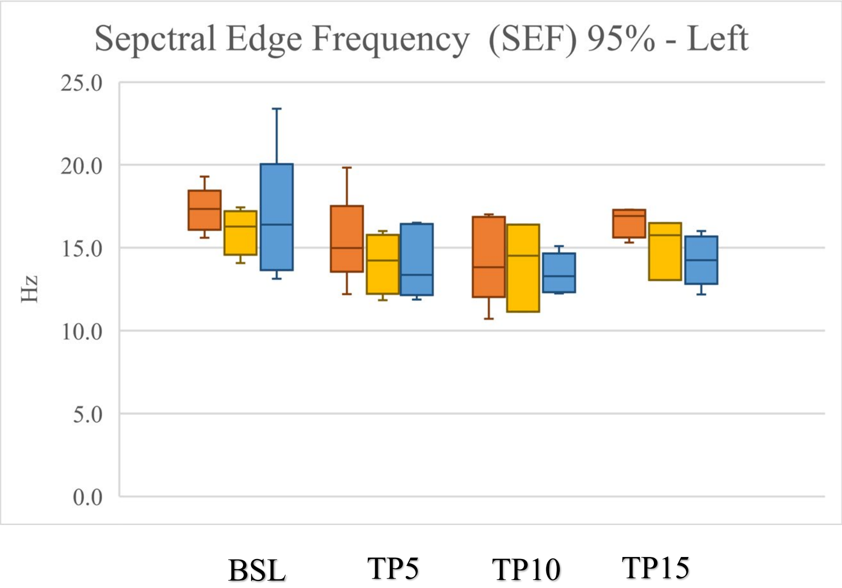

| | BSL | TP5 | TP10 | TP15 |
|---|---|---|---|---|
| **Control** | 3.8 | 5.5 | 1.0 | |
| | 1.0 | 4.0 | 5.0 | 4.0 |
| | 0.0 | 0.4 | 0.2 | 1.0 |
| | 0.0 | 0.0 | 0.0 | 0.1 |
| | 0.0 | 0.7 | 0.0 | 1.6 |

| | BSL | TP5 | TP10 | TP15 |
|---|---|---|---|---|
| **MP 10 mg/kg** | 0.7 | 1.3 | 0.8 | 1.8 |
| | | | | |
| | 1.0 | 3.8 | 3.7 | 4.1 |
| | 0.0 | 0.1 | 2.6 | 4.8 |
| | 0.0 | 0.0 | | |

| | BSL | TP5 | TP10 | TP15 |
|---|---|---|---|---|
| **MP 20 mg/kg** | 0.6 | 1.8 | 0.1 | 2.0 |
| | 2.7 | 0.2 | 1.5 | 1.7 |
| | 0.1 | 0.4 | 2.2 | 2.6 |
| | 3.5 | 0.0 | 0.0 | 6.8 |
| | 2.8 | 0.0 | 0.0 | 0.0 |

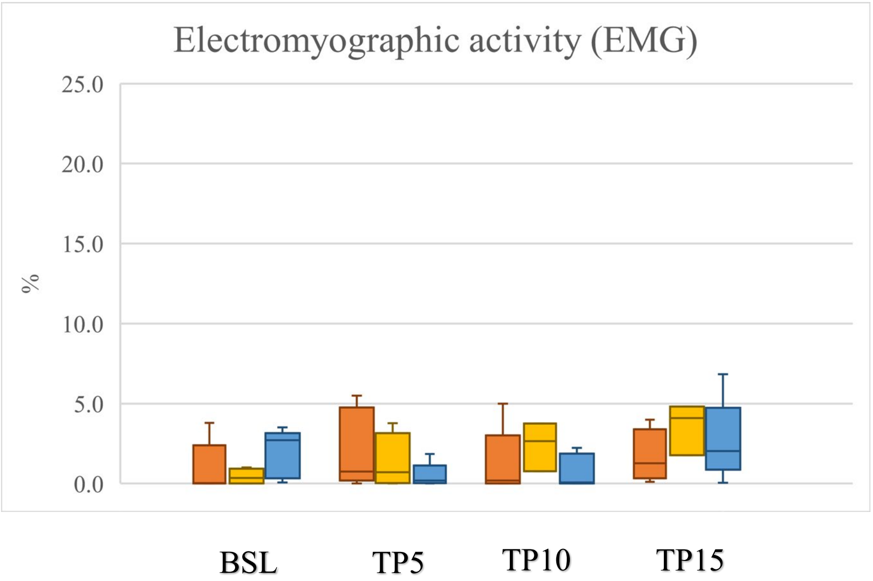

| Control | BSL | TP5 | TP10 | TP15 |
|---|---|---|---|---|
| | 0.0 | 13.2 | 18.7 | |
| | | | | |
| | 0.0 | 0.8 | 0.0 | 0.0 |
| | 4.0 | 0.0 | 0.0 | 0.0 |
| | 0.0 | 0.4 | 0.0 | 0.0 |

| MP 10 mg/kg | BSL | TP5 | TP10 | TP15 |
|---|---|---|---|---|
| | 1.0 | 0.0 | 0.0 | 0.0 |
| | | | | |
| | 0.0 | 0.0 | 0.0 | 0.0 |
| | 0.0 | 0.5 | 0.0 | 0.0 |
| | 0.0 | 0.0 | | |

| MP 20 mg/kg | BSL | TP5 | TP10 | TP15 |
|---|---|---|---|---|
| | 2.8 | 0.9 | 0.4 | 0.7 |
| | 0.0 | 1.2 | 0.0 | 0.0 |
| | 0.9 | 0.0 | 0.0 | 0.0 |
| | 15.8 | 0.0 | 0.0 | 0.0 |
| | 0.4 | 0.0 | 0.0 | 0.0 |

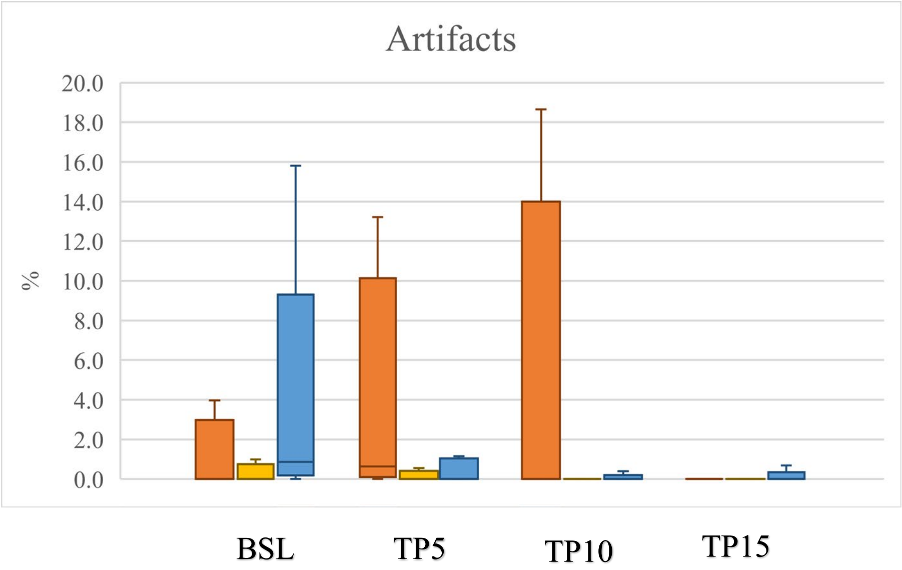

| | BSL | TP5 | TP10 | TP15 |
|---|---|---|---|---|
| **Control** | -3.89856 | 4.359609 | 4.671479 | 6.544087 |
| | -0.82816 | 4.253088 | 5.505154 | 6.951833 |
| | 2.095219 | 9.122862 | 10.70582 | 10.72401 |
| | -4.01078 | 6.359597 | 8.464204 | 5.448457 |
| | -3.98539 | 2.891992 | 5.91848 | 6.816953 |

| | BSL | TP5 | TP10 | TP15 |
|---|---|---|---|---|
| **MP 10 mg/kg** | 1.30351 | 6.107353 | 7.401089 | 7.199638 |
| | 0.953399 | 7.895345 | 8.054252 | 11.9692 |
| | -2.8281 | 5.745578 | 8.228631 | 8.076737 |
| | -2.14032 | 6.170274 | 9.814724 | 9.593459 |
| | 1.538465 | 7.486937 | 8.591409 | 10.06063 |

| | BSL | TP5 | TP10 | TP15 |
|---|---|---|---|---|
| **MP 20 mg/kg** | -0.19515 | 1.751867 | 7.155777 | 8.311639 |
| | 0.641129 | 6.963266 | 7.014211 | 8.007494 |
| | 2.702936 | 9.168995 | 12.86681 | 12.39587 |
| | -6.46764 | 6.965657 | 8.67878 | 5.648243 |
| | -1.95267 | 6.369163 | 7.61756 | 4.006843 |

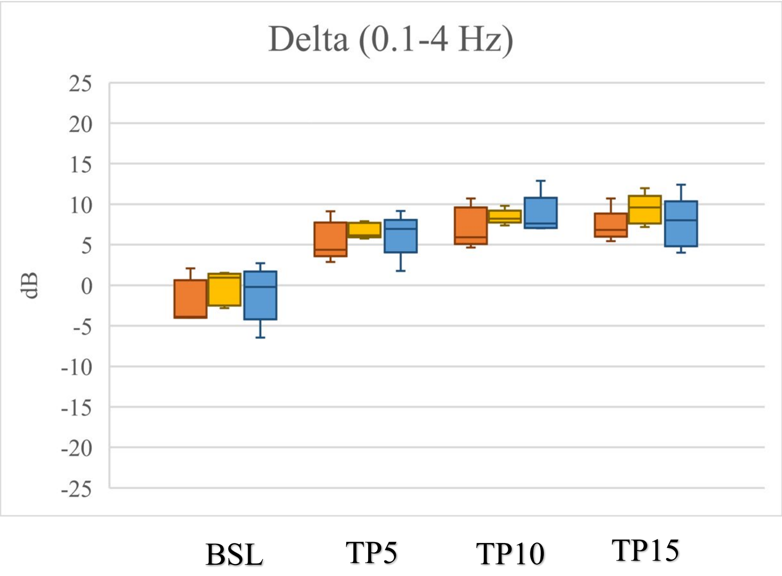

| | BSL | TP5 | TP10 | TP15 |
|---|---|---|---|---|
| **Control** | -4.952711 | 0.970323 | 2.382273 | 1.487645 |
| | -1.823132 | 2.070042 | 3.673999 | 3.946589 |
| | -2.731534 | 3.771711 | 6.10468 | 5.049357 |
| | -7.19818 | 1.992134 | 2.349173 | 0.737261 |
| | -6.415401 | 1.153908 | 3.494659 | 3.252445 |

| | BSL | TP5 | TP10 | TP15 |
|---|---|---|---|---|
| **MP 10 mg/kg** | -2.930354 | 2.19555 | 4.899283 | 5.220578 |
| | -0.686324 | 5.184891 | 5.204897 | 6.259343 |
| | -5.306126 | 1.739379 | 2.211284 | 3.247688 |
| | -4.74439 | 2.366945 | 6.286074 | 5.208795 |
| | -3.030169 | 3.015665 | -0.308936 | 0.944481 |

| | BSL | TP5 | TP10 | TP15 |
|---|---|---|---|---|
| **MP 20 mg/kg** | -2.841867 | -0.502213 | 3.029789 | 5.041209 |
| | -0.64106 | 3.80402 | 5.161401 | 5.012309 |
| | -0.751629 | 5.996837 | 8.292182 | 8.995277 |
| | -13.2649 | 0.020796 | 2.463381 | -2.047862 |
| | -5.118154 | 1.760397 | 1.795279 | -1.207853 |

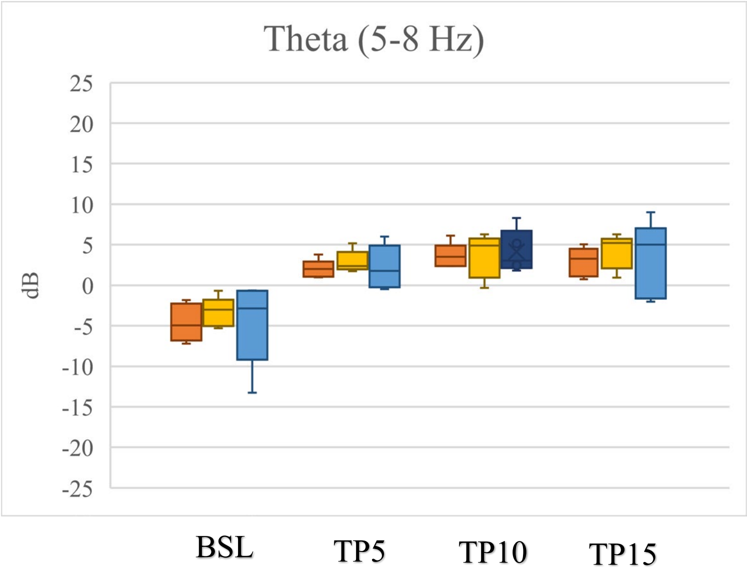

| | BSL | TP5 | TP10 | TP15 |
|---|---|---|---|---|
| **Control** | -8.781388 | -3.10182 | -1.356682 | -0.850595 |
| | -6.128398 | -1.190084 | -0.625307 | 0.073872 |
| | -6.893004 | 0.247599 | 2.078779 | 1.156051 |
| | -9.448398 | -1.297716 | -0.76029 | -0.315524 |
| | -10.26378 | -3.514744 | -0.18517 | 0.198609 |

| | BSL | TP5 | TP10 | TP15 |
|---|---|---|---|---|
| **MP 10 mg/kg** | -4.837782 | -1.122852 | 0.285847 | 1.230213 |
| | -5.237586 | 0.268628 | 1.529374 | 2.980973 |
| | -8.961801 | -3.016972 | -1.589834 | -0.145006 |
| | -8.08872 | -1.006335 | 1.167415 | 2.792961 |
| | -6.077388 | 0.259059 | -2.167942 | -0.821365 |

| | BSL | TP5 | TP10 | TP15 |
|---|---|---|---|---|
| **MP 20 mg/kg** | -5.69516 | -4.264025 | -0.975453 | 0.543575 |
| | -5.104848 | -0.900053 | 0.464697 | 1.635885 |
| | -4.600176 | -0.081826 | 4.02736 | 5.717968 |
| | -12.75669 | -4.226836 | -0.448107 | -4.831975 |
| | -11.18204 | -3.406721 | -1.575486 | -2.879306 |
| | | | | |

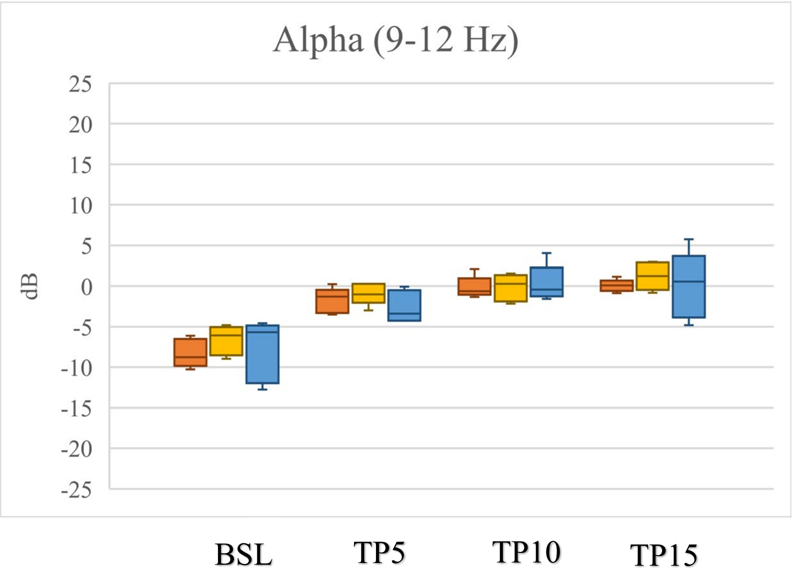

| | BSL | TP5 | TP10 | TP15 |
|---|---|---|---|---|
| **Control** | -15.08126517 | -10.58263 | -9.779428 | -8.306993 |
| | -12.80103513 | -8.888036 | -8.199823 | -8.307228 |
| | -12.63750131 | -6.671932 | -6.450199 | -5.676034 |
| | -14.92447687 | -8.899811 | -7.852123 | -6.432235 |
| | -15.55398227 | -10.59895 | -8.946993 | -8.398775 |

| | BSL | TP5 | TP10 | TP15 |
|---|---|---|---|---|
| **MP 10 mg/kg** | -11.73370621 | -8.268573 | -7.63259 | -7.430463 |
| | -11.61415244 | -6.539868 | -6.984966 | -4.429073 |
| | -15.58744342 | -9.43287 | -9.245369 | -8.061135 |
| | -14.46798216 | -7.93535 | -6.458121 | -5.580108 |
| | -13.6287687 | -7.303692 | -4.428689 | -4.668904 |

| | BSL | TP5 | TP10 | TP15 |
|---|---|---|---|---|
| **MP 20 mg/kg** | -12.05258219 | -11.03243 | -8.311548 | -7.674018 |
| | -11.7821623 | -8.291192 | -7.353024 | -5.924306 |
| | -11.62500551 | -6.694221 | -3.861559 | -1.611978 |
| | -18.1615593 | -11.32418 | -8.150344 | -7.3463 |
| | -16.84374627 | -10.66239 | -7.590162 | -4.924318 |

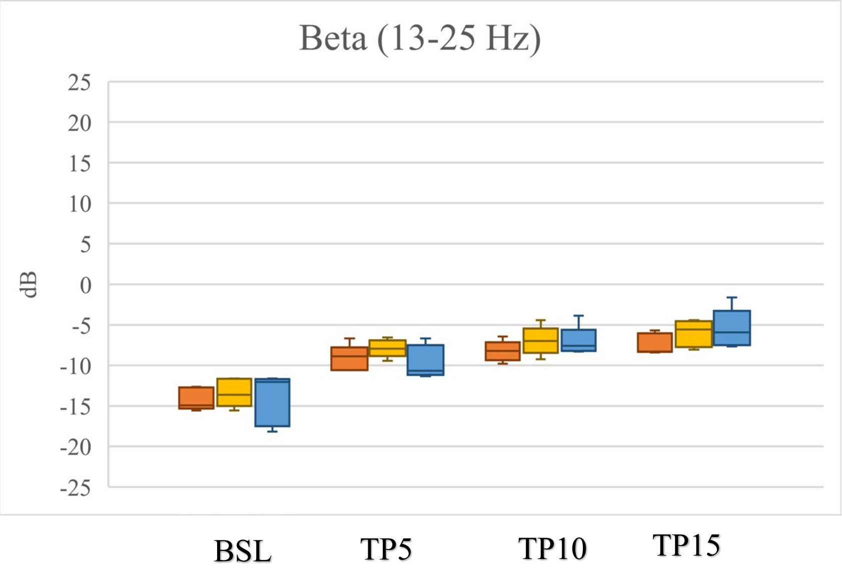

| | BSL | TP5 | TP10 | TP15 |
|---|---|---|---|---|
| **Control** | -22.90346877 | -20.49534418 | -20.7666935 | -17.49803228 |
| | -21.51479739 | -19.49214534 | -18.82280332 | -18.32207261 |
| | -20.75483988 | -17.35751517 | -16.47363319 | -15.25321405 |
| | -24.03914682 | -20.35271691 | -18.7492548 | -16.52882056 |
| | -23.03501831 | -21.17354472 | -19.25478404 | -18.03054195 |

| | BSL | TP5 | TP10 | TP15 |
|---|---|---|---|---|
| **MP 10 mg/kg** | -19.27389527 | -18.92294775 | -18.00170069 | -16.8910742 |
| | -21.64091198 | -17.02638765 | -17.19504092 | -13.51905516 |
| | -23.42947014 | -20.28652692 | -19.09329499 | -18.24304825 |
| | -21.38332849 | -18.70206842 | -16.93071108 | -15.62635934 |
| | -22.6237589 | -17.07811671 | -13.15704126 | -12.55195938 |

| | BSL | TP5 | TP10 | TP15 |
|---|---|---|---|---|
| **MP 20 mg/kg** | -21.83179287 | -20.91990916 | -19.04239314 | -18.40920354 |
| | -21.17424099 | -18.74513889 | -18.67037693 | -16.35156023 |
| | -19.98032548 | -16.75794678 | -14.37520803 | -11.99813937 |
| | -24.09640711 | -21.61253458 | -18.01716456 | -14.42907795 |
| | -23.25448421 | -20.71509571 | -16.78704907 | -13.40317668 |

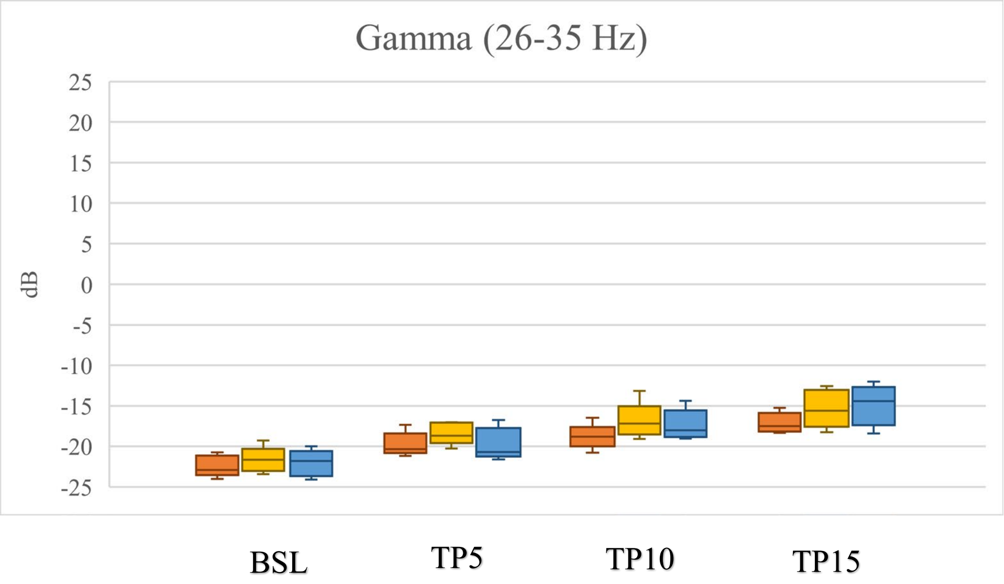

|  | BSL | TP5 | TP10 | TP15 |
|---|---|---|---|---|
| **Control** | -13.87556 | -9.145987 | -8.537215 | -6.880628 |
|  | -11.59745 | -7.935574 | -7.086256 | -6.677046 |
|  | -11.10717 | -5.517186 | -4.509055 | -4.107748 |
|  | -14.47361 | -7.906918 | -6.691489 | -6.110501 |
|  | -14.4221 | -9.569587 | -7.389677 | -6.711126 |

|  | BSL | TP5 | TP10 | TP15 |
|---|---|---|---|---|
| **MP 10 mg/kg** | -10.27311 | -7.274816 | -6.152631 | -5.658484 |
|  | -10.73116 | -5.388649 | -5.431851 | -2.66559 |
|  | -14.1151 | -8.385418 | -7.430383 | -6.511967 |
|  | -12.88753 | -7.052401 | -4.843753 | -4.134186 |
|  | -12.00415 | -5.977655 | -4.33594 | -3.761659 |

|  | BSL | TP5 | TP10 | TP15 |
|---|---|---|---|---|
| **MP 20 mg/kg** | -11.39237 | -10.08129 | -7.068828 | -6.109915 |
|  | -10.68579 | -6.911241 | -6.242157 | -4.830593 |
|  | -9.967246 | -5.141648 | -2.23167 | -0.558466 |
|  | -17.04136 | -9.592654 | -6.518642 | -6.640856 |
|  | -14.62438 | -8.90282 | -6.321545 | -5.398953 |

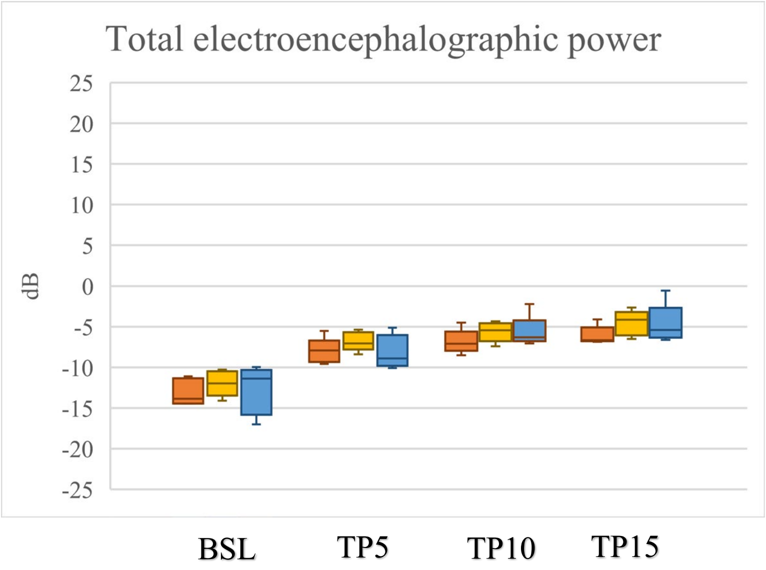

Supplement: S1 Appendix — The blank spaces correspond to missing values. TP: time point; MP: methylphenidate; BSL: baseline; PSI: patient state index; NWR: nociceptive withdrawal reflex. (PDF) [file pone.0302166.s001.pdf]
