# Supplementary material for: The effect of methylphenidate on anaesthesia recovery: An experimental study in pigs
Source: PLoS One. 2024 Apr 16;19(4):e0302166. doi: 10.1371/journal.pone.0302166 (PMC11020859; doi:10.1371/journal.pone.0302166)

	BSL	TP5	TP10	TP15
**Control**	104	136	139	134
	136	143	134	130
	120	120	120	120
	136	150	151	151
	149	126	126	120
	129	144	171	173
	103	119	114	109
	101	124	141	128

	BSL	TP5	TP10	TP15
**Methylphenidate**	133	144	126	118
	142	140	140	140
	135	142	128	124
	104	110	121	125
	134	200	180	180
	110	101	96	117
	207	114	115	115
	153	170	172	170

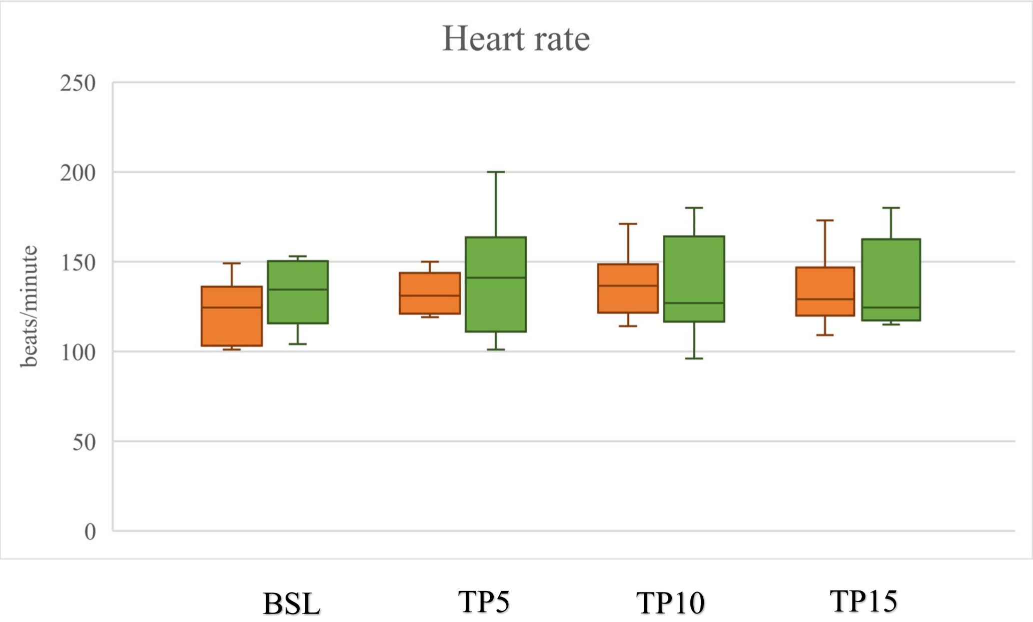

	BSL	TP5	TP10	TP15
**Control**	x	20	17	17
	x	28	28	28
	x	28	28	28
	x	40	36	36
	x	30	32	38
	x	32	32	32
	x	20	20	20
	x	80	80	80

	BSL	TP5	TP10	TP15
**Methylphenidate**	x	20	30	30
	x			
	x	32	30	30
	x	28	30	30
	x	48	48	44
	x	20	20	20
	x	60	60	60
	x	48	48	48

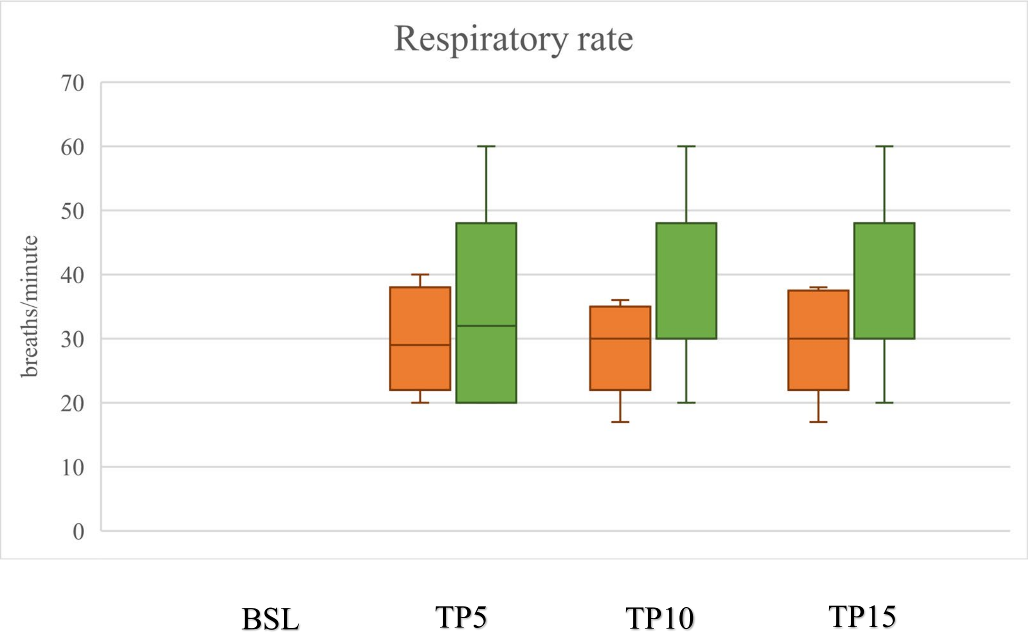

	BSL	TP5	TP10	TP15
Control	68	59	63	59
	105	100	95	100
	87	96	96	72
	72		63	67
	97	78	62	63
	103	109	124	87
	65	70	54	71

	BSL	TP5	TP10	TP15
Methylphenidate	91	90	78	78
	107	96	94	94
	86	110	100	90
	129	103	90	79
	96	117	69	70
	92	108	90	86
	108	116	110	112
	88	80	73	70

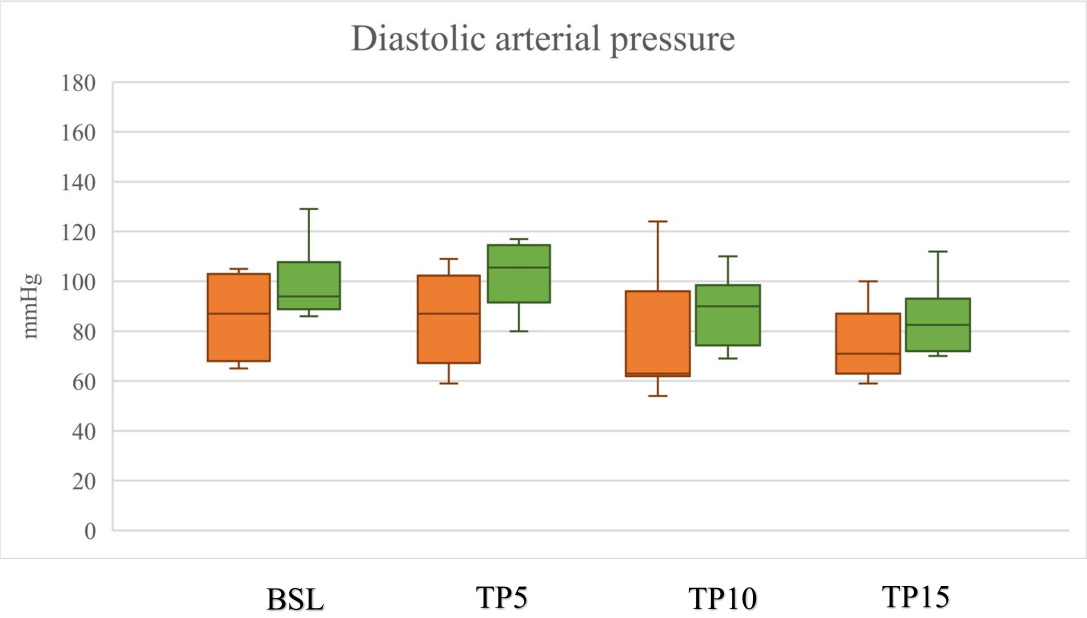

	BSL	TP5	TP10	TP15
**Control**	90	79	81	76
	122	110	108	115
	101	114	95	91
	92		81	86
	114	99	84	84
	117	125	134	104
	84	96	74	92

	BSL	TP5	TP10	TP15
**Methylphenidate**	104	106	92	89
	121	105	104	105
	104	128	120	109
	131	108	105	97
	102	138	88	87
	113	127	125	125
	126	136	136	127
	99	97	93	80

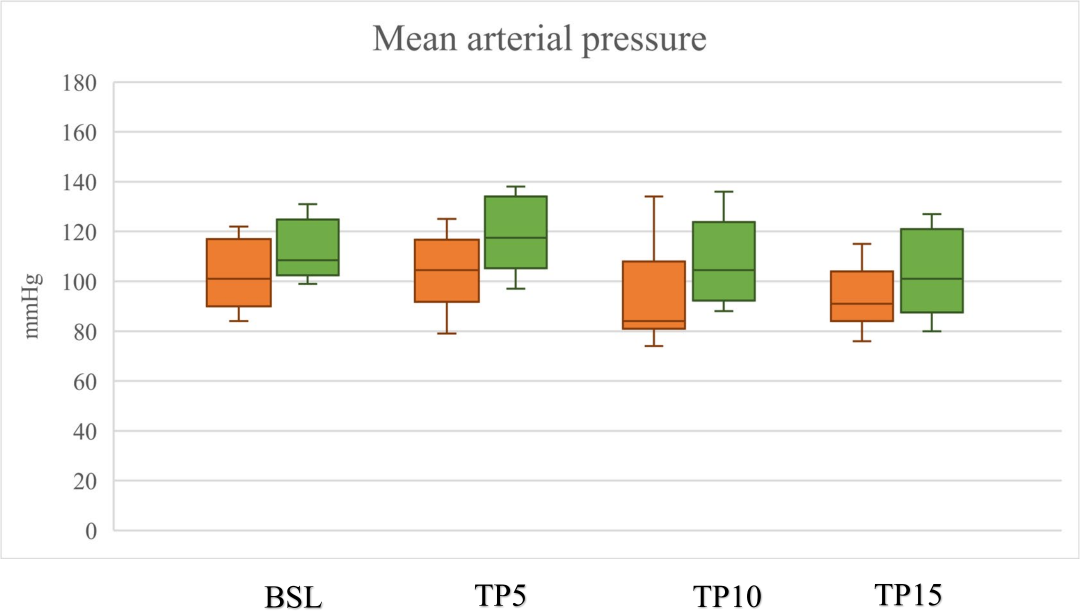

	BSL	TP5	TP10	TP15
**Control**	117	102	102	96
	144	130	132	135
	119	135	132	130
	124		110	109
	145	133	115	117
	132	157	152	133
	107	112	141	130

	BSL	TP5	TP10	TP15
**Methylphenidate**	118	118	107	101
	135	115	120	119
	134	161	150	140
	134	106	127	118
	125	171	120	111
	140	154	146	125
	155	170	170	168
	127	124	117	110

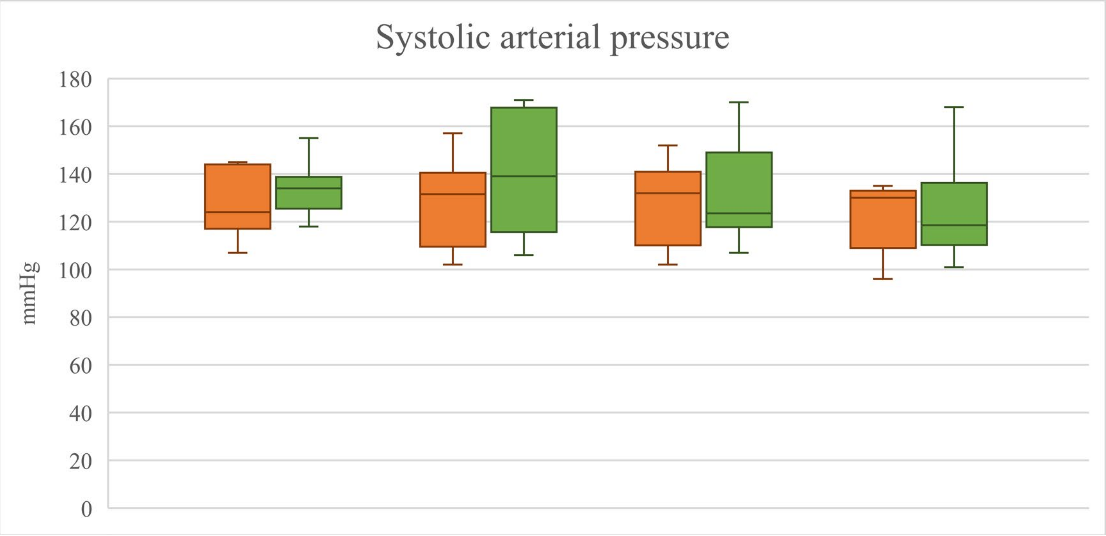

BSL TP5 TP10 TP15

	PSI 70	PSI 80
**Control**		
	34	74
	41	x
		46
	4	29
	6	15
	15	23

Already present

	PSI 70	PSI 80
**Methylphenidate**	32	57
	3	14
	5	11
	2	15
		8
		13
	56	
	2	58

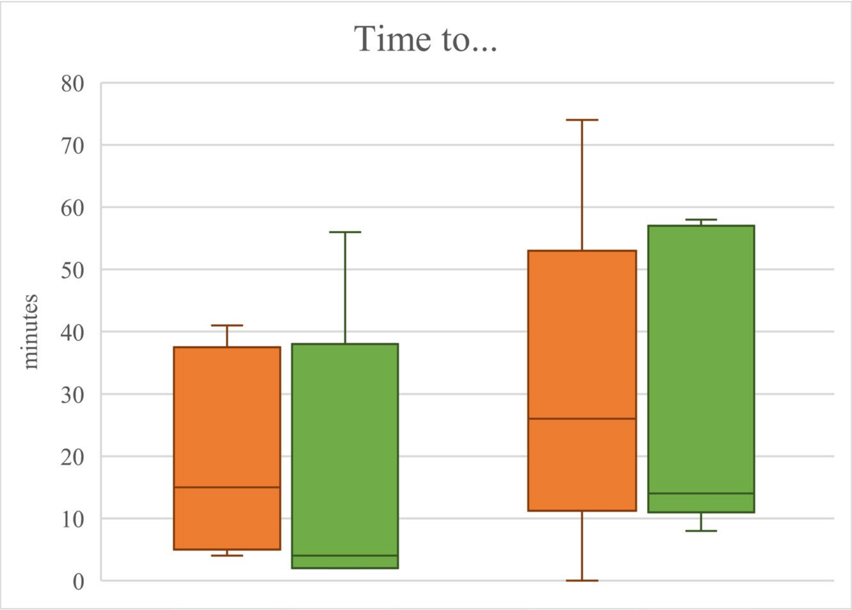

PSI 70 PSI 80

	BSL	TP5	TP10	TP15
**Control**	27.0	45.0		50.0
	40.0	54.0	56.0	60.0
	46.0	54.0	65.0	64.0
	57.4	73.1	72.8	79.5
	45.4	70.1	77.2	77.2
	50.3	75.3	76.0	81.5
	40.0	63.9	63.1	69.6
	76.5	73.0	67.3	63.8

	BSL	TP5	TP10	TP15
**Methylphenidate**	46.0	48.0	51.0	62.0
	41.5	68.2	78.4	81.5
	39.0	73.3	80.1	81.2
	42.4	78.3	79.2	82.0
	54.6	75.0	80.7	72.2
	46.3	75.9	78.9	82.8
	55.3	52.5	52.1	52.5
	56.9	77.9	74.0	73.0

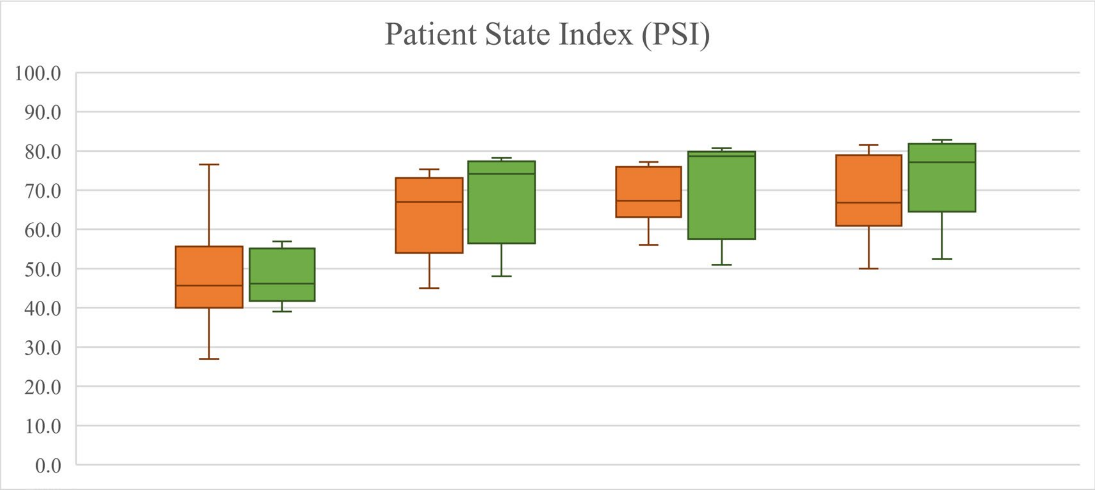

BSL TP5 TP10 TP15

	BSL	TP5	TP10	TP15
Control	11.5	13.9		15.0
	16.0	16.0	17.0	
	16.4	15.6	21.2	21.2
	17.9	22.5	23.6	21.5
	14.8	19.4	21.4	24.6
	12.0	21.2	19.6	18.4
	14.2	20.2	19.9	20.0
	24.3	19.9	19.7	18.6

	BSL	TP5	TP10	TP15
Methylphenidate	15.1	19.7	17.4	22.5
	13.1	22.5	22.5	25.0
	11.9	23.4	19.3	18.3
	14.4	22.5	5.9	3.9
	16.0	22.7	22.0	7.9
	14.4	24.6	22.1	12.2
	14.4	14.6	14.7	16.0
	16.7	24.8	22.3	20.1

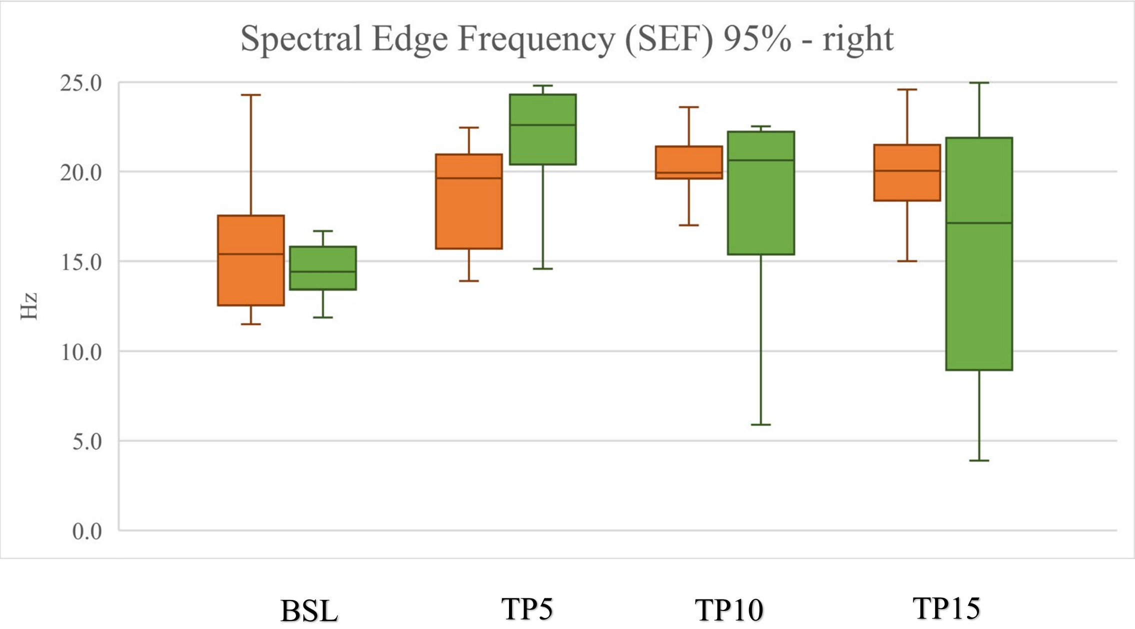

	BSL	TP5	TP10	TP15
**Control**	10.5	13.6		17.2
	14.0	16.0	15.0	
	11.2	19.9	21.6	10.7
	17.0	22.1	21.2	20.4
	15.4	21.6	23.6	24.5
	11.3	23.2	20.5	22.9
	13.6	19.3	17.6	21.6
	24.0	18.8	19.8	18.9

	BSL	TP5	TP10	TP15
**Methylphenidate**	12.5	17.6	16.5	22.1
	11.2	22.2	20.4	24.4
	12.9	21.8	19.4	20.1
	13.2	23.4	11.9	3.3
	15.7	23.2	22.3	22.3
	16.3	24.8	24.1	20.8
	13.8	14.2	15.1	16.6
	16.8	25.5	21.2	19.5

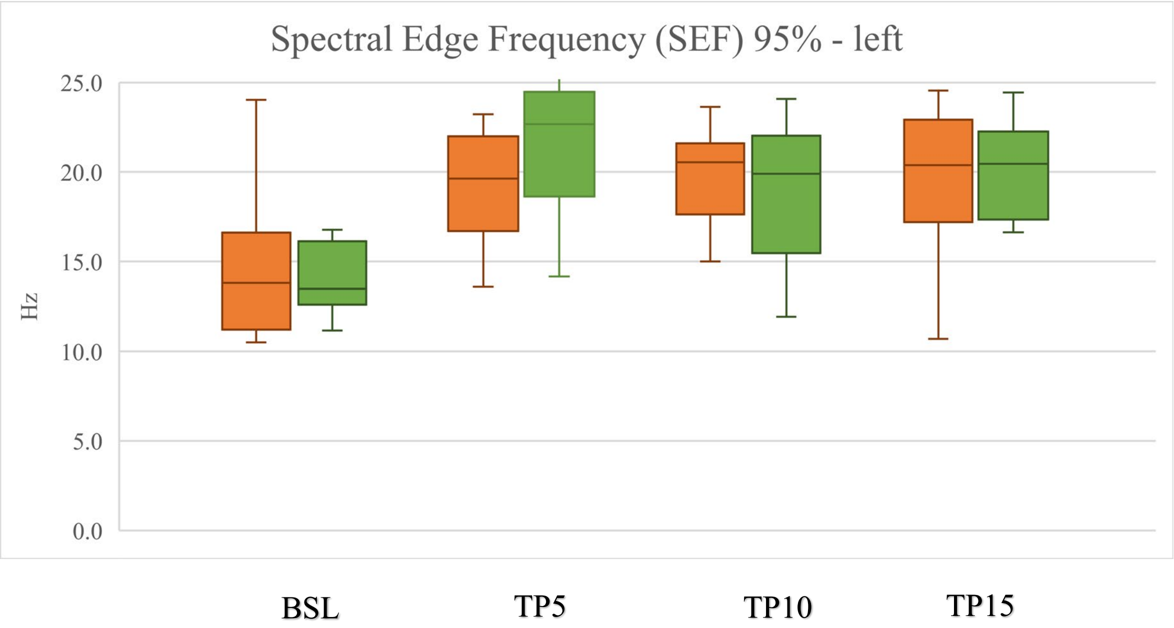

	BSL	TP5	TP10	TP15
**Control**	3.0	3.0		4.0
	6.0	1.0	7.0	9.0
	0.0	5.0	5.0	7.0
	7.9	6.6	7.1	22.6
	2.4	3.2	10.5	14.5
	4.8	4.9	4.8	13.2
	5.5	3.7	3.4	5.7
	15.6	2.6	6.2	6.0

	BSL	TP5	TP10	TP15
**Methylphenidate**	8.0	8.0	10.0	10.0
	6.3	11.5	10.2	23.1
	0.0	11.5	14.3	15.8
	11.8	16.8	15.4	14.3
	5.6	9.2	22.7	21.2
	11.0	8.9	14.4	24.6
	0.0	0.1	0.0	0.0
	0.4	16.0	7.4	9.0

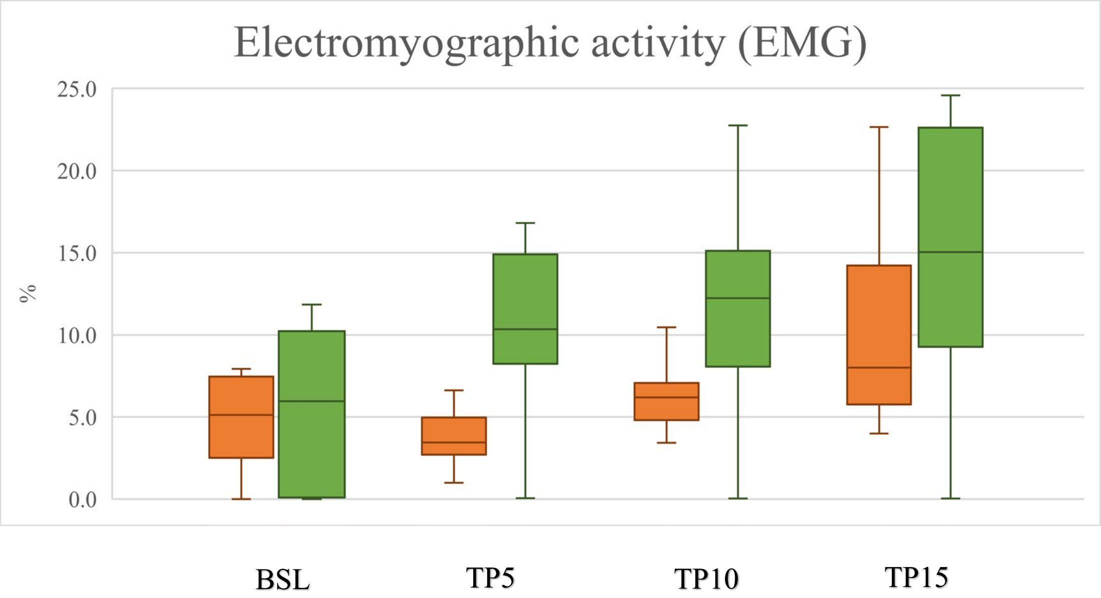

	BSL	TP5	TP10	TP15
**Control**				
	0.0	0.0	0.0	0.5
	0.0	0.0	0.0	0.0
	0.0	0.0	1.8	0.0
	19.7	0.0	0.0	0.0
	3.0	0.6	0.0	0.0

	BSL	TP5	TP10	TP15
**Methylphenidate**				
	0.0	6.1	34.0	52.8
	0.0	0.0	0.0	0.0
	0.0	0.0	20.6	37.9
	0.0	0.0	0.1	3.7
	0.0	2.7	0.3	10.1
	0.0	0.0	0.0	0.0
	0.0	0.0	0.0	0.0

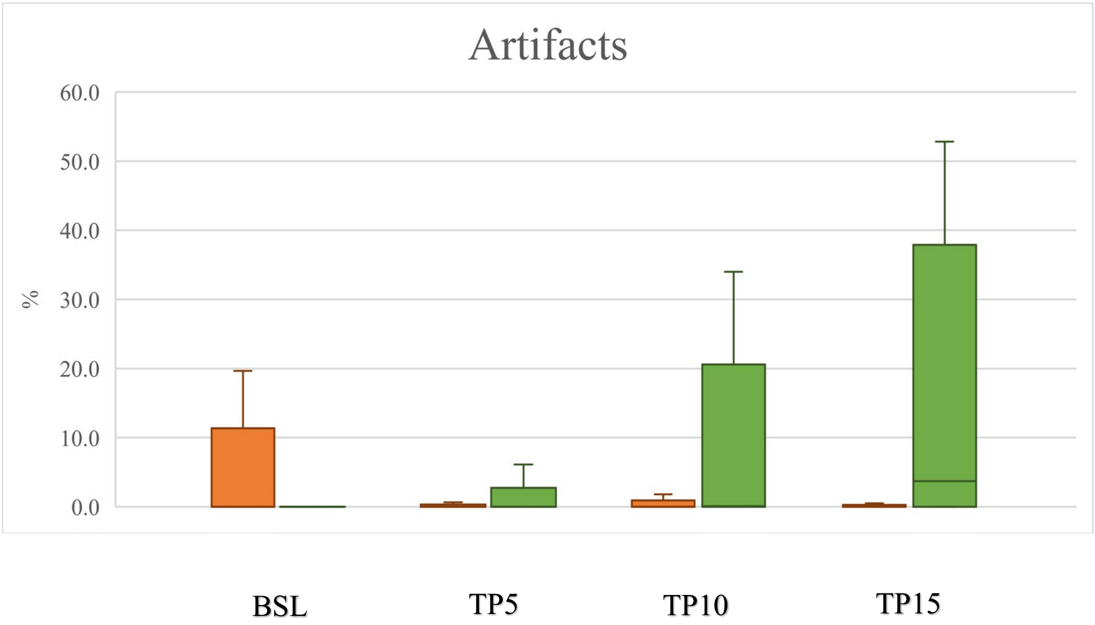

	BSL	TP5	TP10	TP15
**Control**	12.62	11.32857	9.25	5.8
	4.375	1.166667	1.3	1.6
	3.8			0.983333
	23.375	24.2	22.06667	19.28
	14.8	11.58333		6
	25.62857	35.04	27.8	18.96667
	3.85	10.78333	12.96667	12.06667
		22.7	22.125	22.83333

	BSL	TP5	TP10	TP15
**Methylphenidate**	2.88		2.85	
	8	10.26	8.14	7.35
	6.45	3.642857	2.857143	4.8
	13.97143	14	12.07143	8.08
	5.06	5.757143	5.771429	4.525
	1.0	1.0	1.0	1.0
	4.771429	3.866667	8.25	4.785714

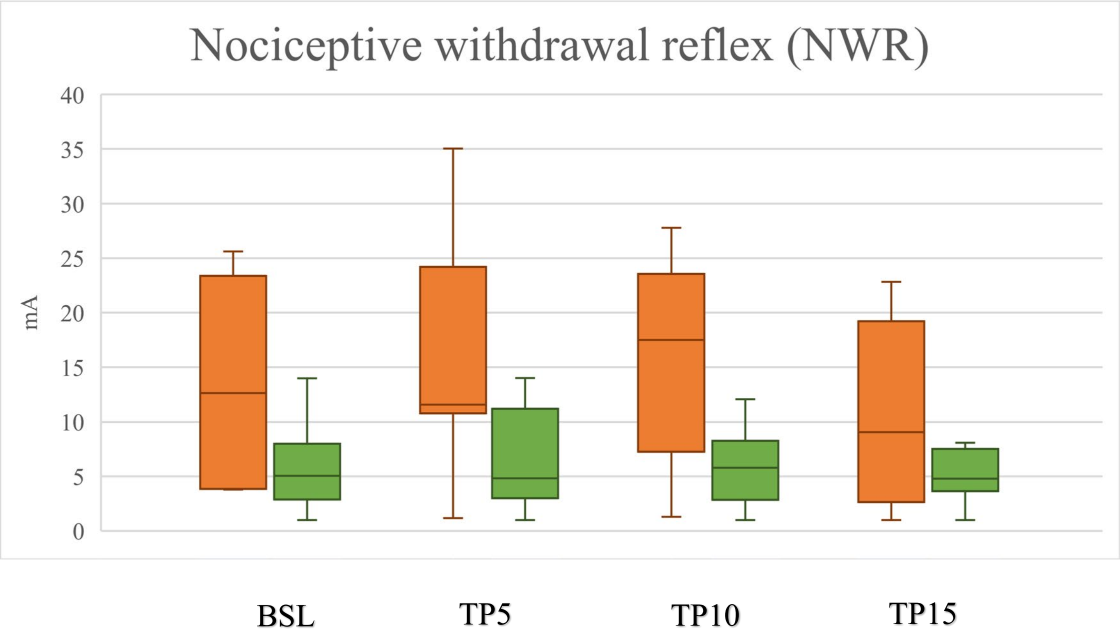

	BSL	TP5	TP10	TP15
**Control**	10.05412	10.09815	9.775334	10.37916
	4.516924	9.511634	7.55233	7.521195
	7.498981	8.279191	7.436877	5.547899
	2.870694	3.296777	0.047951	-1.329278
	5.79179	4.522793	4.449772	1.692397
	4.999151	4.434455	3.112575	2.345355
	9.815451	7.91793	8.95342	9.694972
	8.558309	6.468842	12.12998	11.85214

	BSL	TP5	TP10	TP15
**Methylphenidate**	8.484118	11.20113	9.939168	8.504309
	8.14833	8.781213	13.34841	7.349011
	5.333168	3.907414	4.045257	6.959882
	5.517839	4.144271	4.763948	15.03074
	0.113105	3.541495	12.07828	11.67911
	4.20409	8.69595	7.994639	15.94318
	3.441969	5.639382	4.895301	3.614777
	5.793542	2.794438	5.772888	6.881125

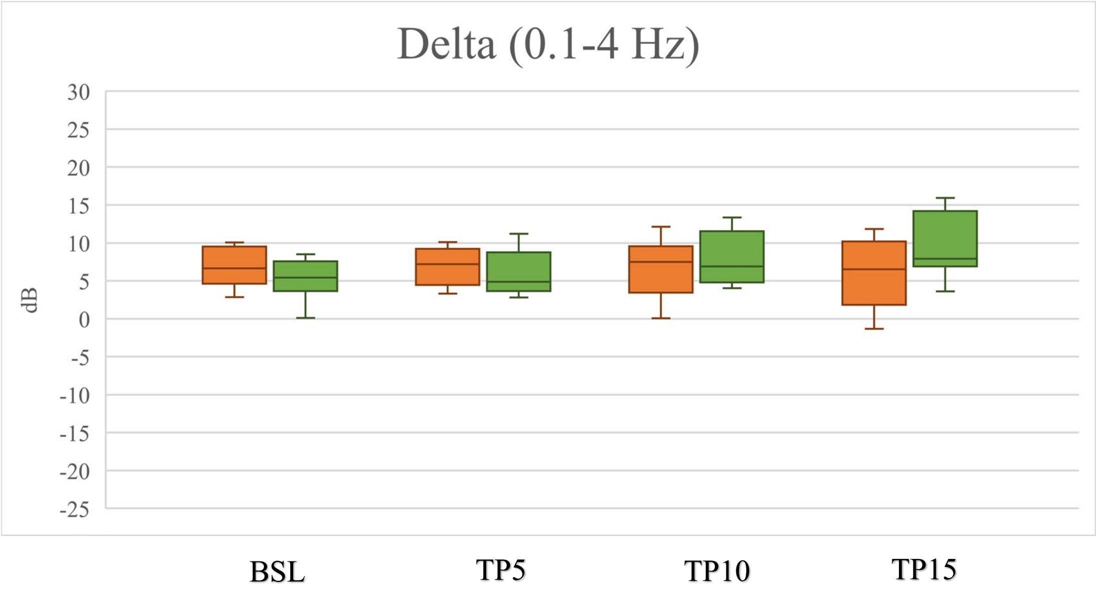

	BSL	TP5	TP10	TP15
**Control**	4.385403	6.289896	6.171879	7.386877
	3.471397	5.632104	3.300438	3.900422
	5.813116	4.664768	4.711775	3.800877
	-1.338264	-4.776159	-6.233683	-7.19572
	1.849711	-2.761693	-3.633258	-5.332131
	0.311953	-2.119327	-2.578285	-4.820305
	5.835997	3.794971	4.306728	4.316243
	1.388267	-0.208284	4.553348	3.715452

	BSL	TP5	TP10	TP15
**Methylphenidate**	4.804523	7.29972	5.977095	5.628405
	3.120327	1.806864	6.664777	1.821961
	0.19073	-1.61484	-3.5896	-1.40771
	1.704263	-1.585151	-0.929845	1.68444
	-3.722505	-3.913057	0.709207	-0.947377
	2.638263	0.337563	1.881253	6.784545
	-0.829387	-0.066141	-0.810536	-0.620492
	1.569003	-1.706186	-0.687664	-1.509388

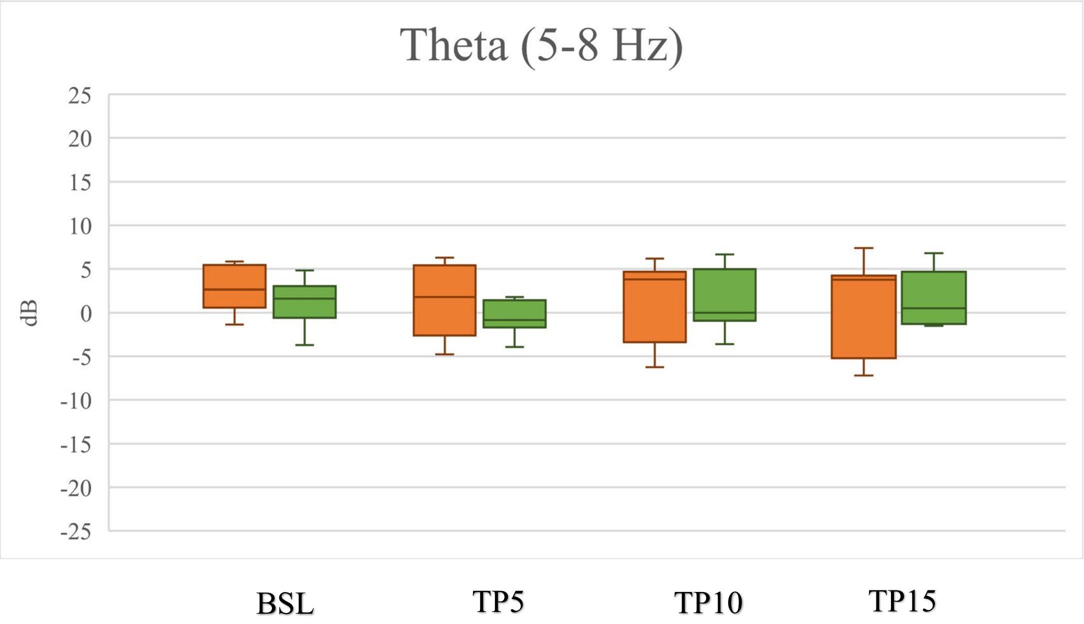

	BSL	TP5	TP10	TP15
**Control**	1.064655	2.41204	1.478163	2.019361
	-1.154209	1.428742	-0.308401	1.635573
	2.173145	1.496538	0.493504	-0.399171
	-5.007356	-8.275462	-7.057038	-9.008494
	-1.868546	-2.795159	-4.16667	-4.889458
	-3.537297	-3.201056	-3.187107	-4.003682
	2.739056	1.708757	1.551764	0.565733
	-2.794894	-1.77374	-2.670867	-1.449224

	BSL	TP5	TP10	TP15
**Methylphenidate**	-0.145419	2.474392	3.127139	1.757563
	-0.119685	0.937332	4.53176	0.459675
	-4.047876	-3.111531	-4.377548	-3.209451
	-1.747235	-2.237414	-3.936682	-2.800954
	-5.101948	-4.721531	-0.3896	-2.908397
	-1.586041	-0.915336	-1.471111	1.759509
	-4.900242	-2.698739	-3.904797	-3.634793
	-1.600823	-2.662661	-2.26867	-3.730884

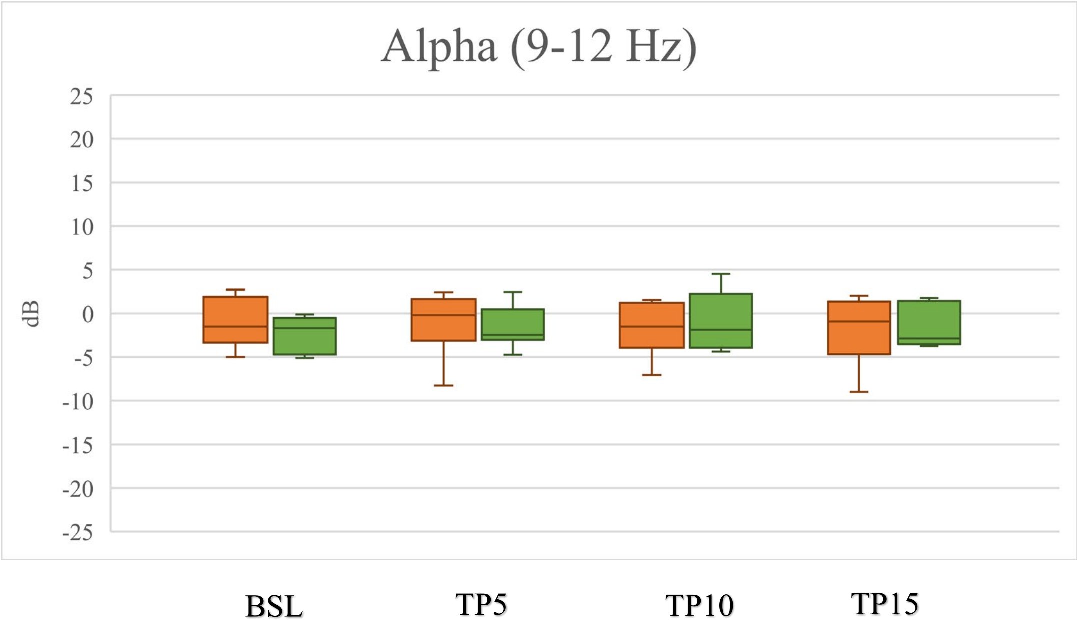

	BSL	TP5	TP10	TP15
**Control**	-7.579836	-4.143515	-4.191949	-3.189092
	-8.908774	-2.966659	-7.054894	-5.344835
	-4.663303	-5.887225	-5.593448	-6.922199
	-10.64207	-10.47067	-10.53721	-9.177957
	-8.739114	-8.832745	-7.652073	-6.939381
	-11.22627	-10.50303	-9.76551	-9.34001
	-5.747046	-2.579062	-2.696087	-3.661278
	-6.620492	-6.596476	-7.909981	-7.380938

	BSL	TP5	TP10	TP15
**Methylphenidate**	-7.84224	-3.840013	-3.732785	-3.655069
	-8.21691	0.115671	3.184187	-0.719834
	-11.79601	-5.106488	-5.531535	-4.765381
	-9.208981	-3.846917	-5.852172	-4.740298
	-11.92381	-10.47385	-1.983491	-4.442898
	-9.018548	-1.828404	-2.291514	-1.043132
	-10.62672	-9.647249	-9.782658	-9.393214
	-8.29974	-4.639546	-5.400525	-6.454487

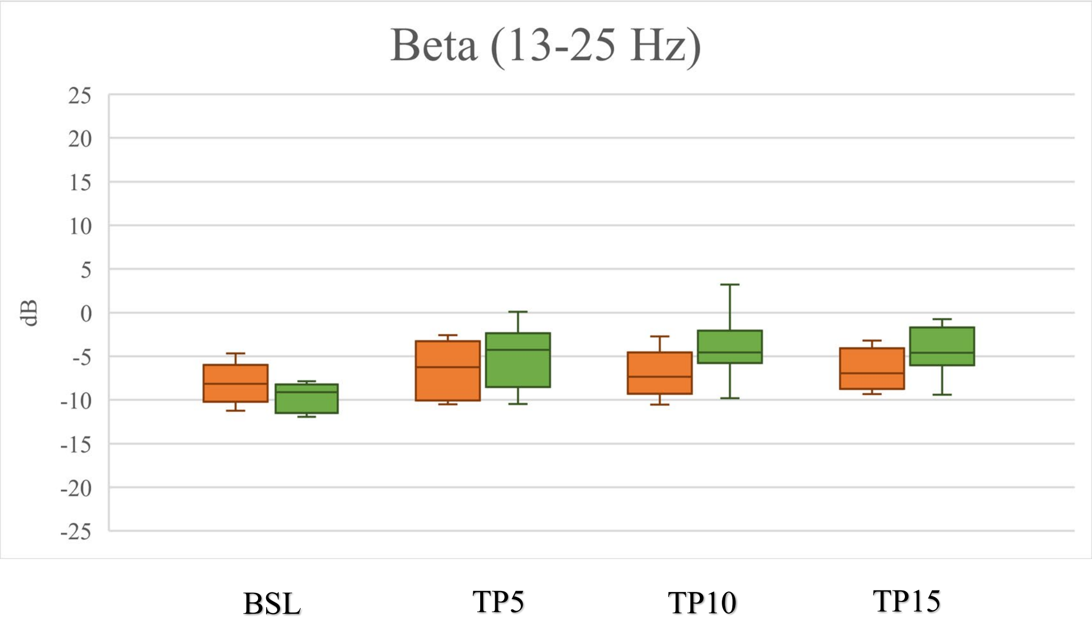

	BSL	TP5	TP10	TP15
**Control**	-16.07321	-14.44427	-13.24903	-11.46807
	-19.14249	-9.1168	-15.52464	-14.48424
	-12.385	-14.9317	-15.18403	-15.75962
	-17.72228	-17.18655	-18.35961	-16.62826
	-19.78942	-17.35803	-15.14716	-15.37379
	-20.76498	-18.99299	-17.22285	-15.51425
	-13.82488	-12.20881	-12.81192	-11.87229
	-13.07175	-13.0666	-15.23398	-15.18636

	BSL	TP5	TP10	TP15
**Methylphenidate**	-16.49873	-14.83514	-13.97699	-13.69605
	-18.65029	-9.915909	-3.854358	-8.814249
	-20.63069	-13.02674	-12.26665	-11.52883
	-16.85741	-11.73847	-12.11002	-10.87619
	-18.80134	-15.80478	-8.022739	-10.74188
	-17.19836	-10.55826	-10.42316	-8.058681
	-19.05067	-18.72482	-19.57609	-19.04021
	-17.45468	-12.25951	-14.72547	-14.74631

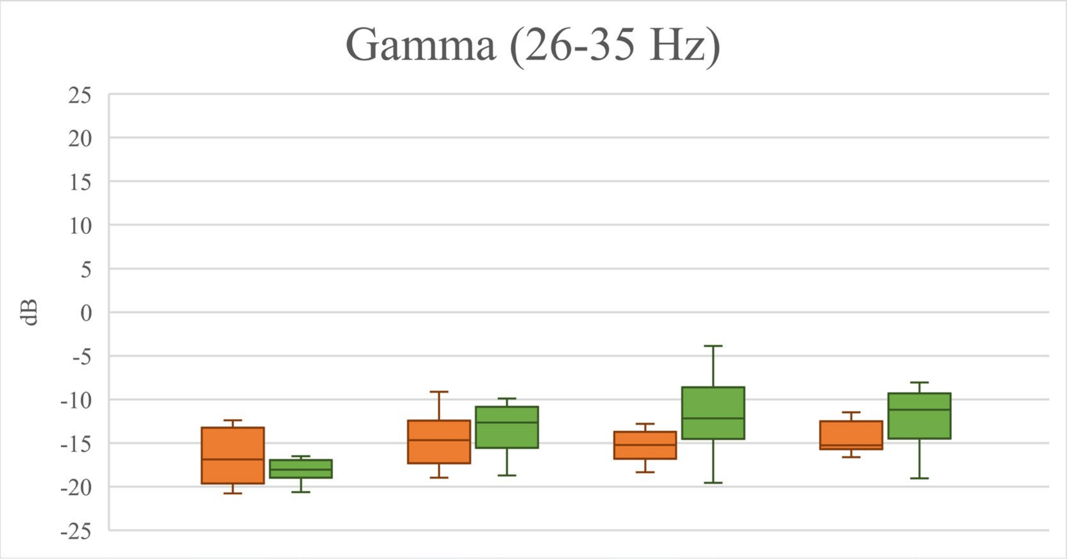

BSL TP5 TP10 TP15

	BSL	TP5	TP10	TP15
**Control**	-5.199976	-3.139164	-2.986355	-1.850504
	-7.6496	-1.498139	-5.478618	-4.293757
	-3.19536	-4.43916	-4.626378	-5.728845
	-9.072187	-9.547411	-10.37508	-9.918313
	-7.850529	-8.000539	-7.219428	-7.677062
	-9.486026	-9.043848	-8.518855	-8.336995
	-3.599526	-2.611427	-2.63798	-2.731021
	-4.989414	-5.333447	-5.194161	-4.985839

	BSL	TP5	TP10	TP15
**Methylphenidate**	-5.718873	-2.865818	-2.838425	-3.122526
	-6.682736	-1.188126	3.177196	-1.434131
	-9.678273	-5.445006	-5.7283	-4.469652
	-7.246488	-4.498987	-5.356244	-2.769392
	-10.4932	-8.639965	-1.231749	-3.394555
	-7.333204	-2.449533	-2.566876	0.54846
	-9.287854	-8.209029	-8.814454	-8.651698
	-7.044527	-5.178121	-5.56729	-6.053525

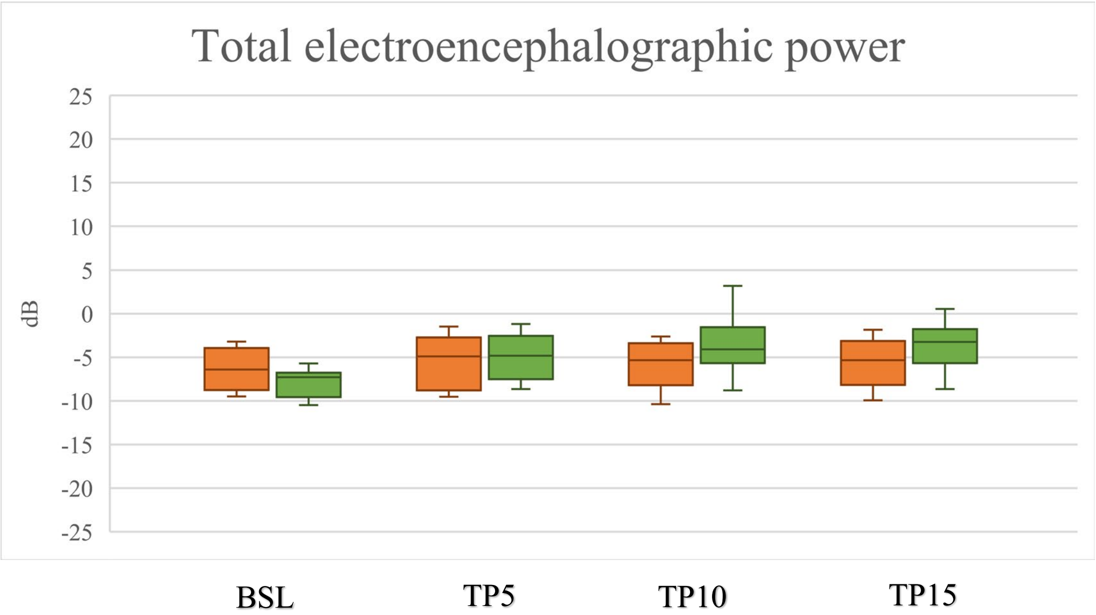

Supplement: S2 Appendix — The blank spaces correspond to missing values. TP: time point; BSL: baseline; PSI: patient state index; NWR: nociceptive withdrawal reflex. (PDF) [file pone.0302166.s002.pdf]
