# Supplementary material for: The effect of methylphenidate on anaesthesia recovery: An experimental study in pigs
Source: PLoS One. 2024 Apr 16;19(4):e0302166. doi: 10.1371/journal.pone.0302166 (PMC11020859; doi:10.1371/journal.pone.0302166)
Supplement: Attachment — Submitted filename: Comments.docx [file pone.0302166.s003.docx]

I congratulate the authors for the detailed and very well conducted study.

I have few questions to better understand the scientific protocol.

1- Why did you choose not to use miorelaxants for tracheal intubation and eventually the antagonist of them?
2- I found the dose of propofol used very high (20mg/kg/h + 4-6mg/kg bolus+ smaller dose boluses+ increased infusion of 6mg/kg/h). Could the use of such a dose of propofol be influenced the efficacy of methylphenidate?
3- In the experiment II you administered the methylphenidate after extubation. How were you able to extubate the pigs without they did not recover completely from sedation?
4- Did you test the CO2 (carbon dioxide)? In order to exclude a high-CO2 related sedation?

Thank you for clarifying.
